# Efficacy of non-invasive brain stimulation in reducing craving in patients with alcohol use disorder: systematic review and meta-analysis

**DOI:** 10.1186/s12888-025-06883-4

**Published:** 2025-05-16

**Authors:** Dae Jin Kim, Hyunsuk Jeong, Su Yeon Kim, Young Hwa Kim, Hyeon Woo Yim

**Affiliations:** 1 Department of Korean Medicine Policy, Korea Institute of Oriental Medicine, Daejeon, Republic of Korea; 2 Department of Preventive Medicine, College of Medicine, The Catholic University of Korea, Seoul, Republic of Korea; 3 Department of Evidence Development Support, National Evidence-Based Healthcare Collaborating Agency, Seoul, Republic of Korea; 4 Divison of Control for Zoonotic and Vector-borne Disease, Korea Disease Control and Prevention Agency, Cheongju, Republic of Korea

**Keywords:** Alcohol use disorder, Craving, Non-invasive brain stimulation, Transcranial direct current stimulation, Repetitive transcranial magnetic stimulation

## Abstract

**Background:**

Craving plays a central role in reinforcing alcohol use disorder (AUD), and non-invasive brain stimulation (NIBS) has shown potential as a therapeutic intervention in AUD. We aim to evaluate the efficacy and safety following the application of NIBS in patients with AUD.

**Methods:**

A search of the PubMed, EMBASE, Cochrane Library and PsycINFO databases for articles published up to June 30, 2024 using predefined search terms identified a total of 20 randomized controlled trials (RCTs) and 22 units. The primary outcome of this study was the change in craving severity. The secondary outcome was the rate of adverse events.

**Results:**

Comparing the effect of alcohol craving severity reduction between the NIBS group and the sham group, the NIBS group showed a significant reduction in alcohol craving severity compared to the sham group (SMD = -0.211; 95% CI = -0.379 to -0.042). The I^2^ value was 22.2%, indicating a low level of heterogeneity (*p* = 0.17). Regarding safety, the NIBS group had an increased rate of adverse events compared to the sham group, but this was not significant (OR = 1.494; 95% CI = 0.834 to 2.675). In a subgroup analysis based on the types of NIBS, only transcranial direct current stimulation showed a significant effect (SMD = -0.214; 95% CI = -0.427 to -0.002). Subgroup analyses of stimulation parameters in NIBS showed that a significant reduction in craving severity was observed when NIBS was applied to the dorsolateral prefrontal cortex (SMD = -0.200; 95% CI = -0.381 to -0.019) and when multiple sessions were administered (SMD = -0.388; 95% CI = -0.620 to -0.156). In addition, a significant reduction in craving severity due to delayed effects was observed even 4 weeks after the last stimulation (SMD = -0.553; 95% CI = -0.979 to -0.126), but this finding should be interpreted with caution.

**Conclusions:**

NIBS is effective in reducing the severity of craving in patients with AUD. This study provides the latest evidence on the effect of NIBS in reducing craving severity in AUD patients.

**Supplementary Information:**

The online version contains supplementary material available at 10.1186/s12888-025-06883-4.

## Introduction

Repeated consumption of alcohol can lead to the development of alcohol use disorder (AUD) [1, 2]. Excessive alcohol consumption is a significant risk to the health of populations around the world. In 2016, alcohol contributed to 3 million fatalities globally, representing 5.3% of all deaths [3]. Alcohol is often used in combination with other substances that are harmful to health [4]. Consequently, it is crucial to urgently develop effective treatments for patients with AUD [5].

Standard treatments for AUD, such as pharmacotherapy and psychotherapy are often limited by poor retention rates and high relapse rates [6, 7]. Recently, non-invasive brain stimulation (NIBS) has emerged as a promising therapeutic option for AUD. NIBS provides a less invasive and cost-effective alternative to invasive brain stimulation techniques. It delivers localized, short-term stimulation to specific brain regions, typically lasting less than 1 h per day, and influences deeper brain regions through functional connectivity, thereby modulating neural activity [8]. Transcranial direct current stimulation (tDCS) and Transcranial magnetic stimulation (TMS) are the most studied methods of NIBS, widely used to modulate neural activity in specific brain regions and promote neuroplasticity [9–12].

Neurobiological changes associated with AUD are complex and not fully understood, though it is well-established that limbic circuits involved in reward processes are heavily engaged. The mesolimbic dopamine pathway plays a central role in craving associated with alcohol dependence, and changes in the reward system due to increased dopamine release from alcohol use reinforce alcohol dependence [13]. Continuous alcohol use alters the sensitivity of the dopamine pathway and disrupts the balance of the reward system, reinforcing both withdrawal symptoms and dependence. Alcohol disrupts normal neurotransmission, particularly by causing persistent activation of GABA-A receptors, which can lead to alcohol tolerance. It also affects the release and reuptake of glutamate, resulting in changes in the neurotransmission system [14, 15].

Craving plays a crucial role in the persistence and treatment of AUD [16]. Clinical neuroimaging studies have identified key regions involved in craving, including the anterior and posterior cingulate cortex, orbitofrontal cortex, insular cortex (IC), medial prefrontal cortex (mPFC), and dorsolateral prefrontal cortex (dlPFC) [17–23]. Various studies have applied NIBS to different brain regions, with the dlPFC being a common target. Stimulation of the dlPFC has been shown to be effective in controlling cravings for substances, including alcohol and nicotine [24–29].

tDCS modulates cortical excitability in the human brain depending on stimulation polarity [30–32]. It can alter cortical excitability with long-lasting effects, offering an alternative for patients who experience side effects or are resistant to medication [33]. Anodal tDCS facilitates depolarization and enhances cortical excitability, whereas cathodal tDCS induces hyperpolarization and reduces excitability at the site of stimulation [34].

TMS is a technique that uses electromagnetic coils to generate magnetic fields that induce electrical activity in the brain, and there are different types of TMS depending on the stimulation method or pulse pattern. Repetitive TMS (rTMS) is a type of TMS that uses repetitive magnetic pulses to influence brain functions altered by substance use, including reward processing, craving, and cognitive control [35, 36]. High-frequency rTMS (HF, ≥ 5 Hz) enhances neural activity and cortical excitability, while low-frequency rTMS (LF, ≤ 1 Hz) is associated with cortical inhibition [37]. The neural effects of rTMS can persist for several minutes post-stimulation and may result in long-term brain activity changes [38]. However, the extent of changes in cortical excitability may vary depending on the intensity of stimulation and the number of pulses delivered per session [11]. The theta burst stimulation (TBS) offers a shorter single session duration with effects similar to rTMS [39, 40].

According to a recent meta-analysis study, both tDCS and TMS did not show effective craving reduction relative to sham [10]. However, the review included non-randomized controlled trials and studies examining combined effects with other treatments, limiting the ability to confirm the specific impact of NIBS on craving reduction. Therefore, this study aims to identify randomized controlled trials (RCTs) that have evaluated the standalone effects of NIBS on craving reduction and safety in patients with AUD and to synthesize the results for quantitative assessment of the impact of NIBS on craving severity.

## Materials and methods

### Compliance with general guidelines

This study complied with the PRISMA 2020 statement (Appendix Table 1). Details of the study protocol are available on the Prospective Register of Systematic Reviews (PROSPERO) website (CRD42024567484).

### Search strategy & eligibility criteria

This study included articles that assessed the efficacy of NIBS in reducing craving severity in patients with AUD, published up to June 30, 2024. Predefined search terms were used to search the PubMed, EMBASE, Cochrane Library and PsycINFO databases. The complete search strategy is detailed in Appendix Tables 2, 3, 4 and 5.

To minimize heterogeneity, we implemented strict inclusion criteria, selecting only RCTs involving patients with AUD. Additional inclusion criteria required that studies apply NIBS to specific brain regions and report results on changes in craving severity. For cross-over studies, only those that implemented a washout period were included in this study.

Exclusion criteria included studies that did not report changes in craving severity, studies without a sham control group, as well as case studies, review articles, and grey literature (e.g., conference abstracts, commentaries, thesis, dissertation and study protocols).

### Data extraction

Two authors (D.J. and Y.H.) independently extracted data using a predefined extraction form. Extracted data included patients’ characteristics (mean age, percentage of women, diagnostic criteria), study characteristics (number of participants per group, treatment duration), stimulation parameters (site of stimulation, number of sessions, stimulation intensity, frequency, electrode size), and outcomes (types of craving assessment tools, craving scores, time point of craving assessment and number of adverse events). According to the Cochrane Handbook, when conducting a meta-analysis that includes studies reporting outcomes at multiple follow-up time points, one suggested approach is to select the longest follow-up period from each study. Therefore, in this analysis, craving scores were extracted at baseline and at the final measurement time point after the last stimulation [41]. We contacted the study authors to obtain essential data that was missing.

### Quality assessment

To assess the quality of the studies, we used the Risk of Bias 1 tool, which assesses sequence generation, allocation concealment, blinding of participants and personnel, blinding of outcome assessment, incomplete outcome data, selective reporting and other biases (Appendix Table 6). The overall risk of bias was classified as low, unclear, or high. The criteria for assessing the overall risk of bias classified studies as low risk if all seven domains under evaluation were rated as low risk or if only one domain was rated as unclear. Studies were classified as unclear risk if two or more domains were rated as unclear. If one or more domains were rated as high risk, the study was classified as high risk. Two authors (D.J. and S.Y.) independently performed the quality assessment and discussed and resolved any discrepancies.

The overall quality of evidence was assessed using GRADEpro GDT. The quality of evidence was graded as high, moderate, low and very low based on an assessment of certainty, including risk of bias, inconsistency, indirectness, imprecision and other considerations.

### Outcome measure

The primary outcome of the study was the change in craving severity. Craving, defined as an intense urge to use a substance causes both physical and psychological discomfort when unmet [42]. Initially considered a withdrawal symptom, craving was recognized as a diagnostic criterion for AUD in the fifth edition of the Diagnostic and Statistical Manual of Mental Disorders [43]. The secondary outcome was the rate of adverse events in participants enrolled in each RCT.

### Statistical analysis

This study was analyzed using the “meta” package in R software (version 4.3.1) [44]. Changes in craving severity were calculated as standardized mean differences (SMDs) with 95% confidence intervals (CIs), and rates of adverse events were calculated as odds ratios (ORs) with 95% CIs. Craving severity was evaluated based on scores recorded at baseline and at the last craving measurement time point. Hedge’s g was calculated using SMDs instead of mean differences (MDs) due to the variation in scales across the included studies [45]. For one study that reported craving severity changes across all four of Lesch’s typology subtypes [46], weighted means and representative standard deviations were computed to reflect the combined results in the meta-analysis. A negative SMD indicates that the NIBS group was more effective in reducing alcohol craving compared to the sham group.

Pooled effect sizes for comparing changes in alcohol craving severity between the NIBS and sham groups were calculated using the “metacont” function in R software [44]. Given the expected variability in participant characteristics and stimulation parameters across the studies, a random-effects (RE) model was used. The RE model assumes that the true effects follow a normal distribution and account for inter-study variation. For three-arm studies, the sample size in the sham group was halved to prevent double counting, in accordance with the Cochrane Handbook [41].

Subgroup analyses were conducted on types of NIBS, stimulation parameters, time point of craving assessment, study design and quality assessment variables to explore the sources of heterogeneity or to assess the impact of potential moderators on the reduction of alcohol craving.

Heterogeneity across studies was assessed using the I^2^ statistic. I^2^ values range from 0 to 100%, with values between 0% and 25% indicating low heterogeneity and values between 25% and 75% suggesting moderate heterogeneity. An I^2^ value of 75% or higher was interpreted as substantial heterogeneity. To assess publication bias, funnel plots and egger’s test were used.

Pooled effect sizes comparing the rate of adverse events between NIBS and sham groups were calculated using the “metabin” function in R software [44]. Studies that did not report event values for both groups were excluded to prevent bias. Additionally, following the Cochrane Handbook, a continuity correction of 0.5 was applied when one group reported zero events to reduce bias [41].

## Results

### Study selection

**Fig. 1:**
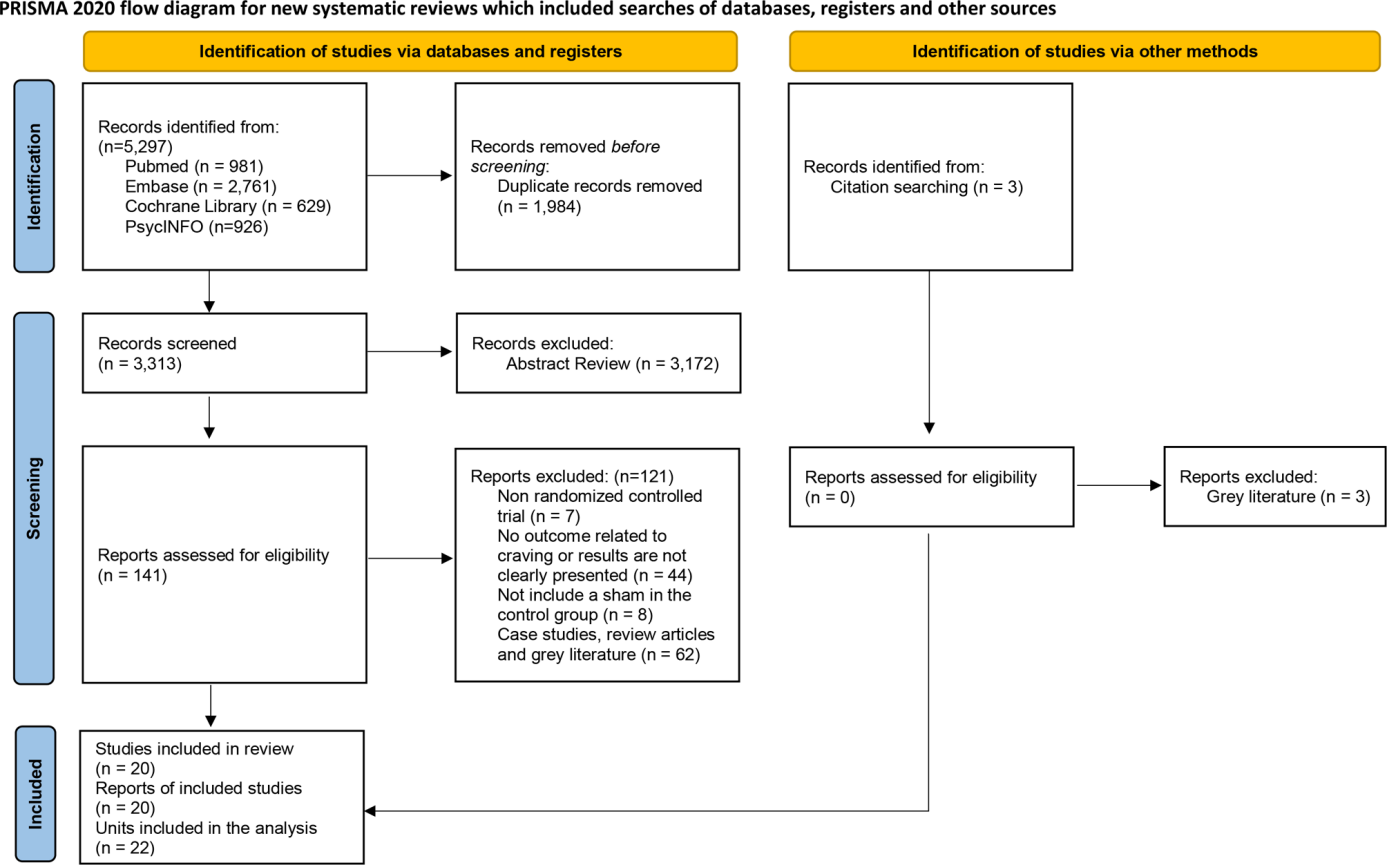
Study selection process

Screening was performed on the titles and abstracts of all articles, excluding those identified as duplicates. As a result, 141 of the initial 5,297 articles identified from the four databases were deemed highly relevant to the study objectives. After a detailed eligibility review of the full-text articles, 20 studies were selected for inclusion. As illustrated in Fig. 1, the PRISMA 2020 flow diagram outlines the study selection process.

### Study characteristics

The meta-analysis included 20 studies comprising 22 units and a total of 860 participants (Table 1). Units refers to pairwise comparisons within each study included in the meta-analysis. Typically, this corresponds to one unit for a two-arm study and two units for a three-arm study. Of the 20 studies, 18 were two-arm studies, and two were three-arm studies. Based on study design, 14 studies were parallel designs, five were cross-over designs and one was a factorial design. For cross-over designs, two studies were identified with a washout period of 24 to 48 h, and three studies had a washout period of 7 to 14 days. All 20 studies were randomized, with six conducted as single-blind trials and 14 as double-blind trials.

**Table 1:** Characteristics of the studies included in the review

Reference	Comparison	Study design	Sample size	Mean age	Female (%)	Stimulation parameters	Duration of treatment	Measurement time point	*N* session	Outcome measure
Boggio et al. (2008) [27]	a-F3 + c-F4 tDCS a-F4 + c-F3 tDCS sham	Ⓒ Ⓡ Ⓓ	13 13 13	41.3 ± 5.7 41.3 ± 5.7 41.3 ± 5.7	15.4 15.4 15.4	2 mA, 20 min	1 day	Pre, Post	1 1 1	AUQ
Mishra et al. (2010) [49]	F4-rTMS sham	Ⓟ Ⓡ Ⓢ	30 15	39.4 ± 8.9 38.2 ± 6.9	0.0 0.0	10 Hz, 110% MT, 1,000 pulses	10 days	Pre, 4 weeks follow up	10 10	ACQ-NOW
Herremans et al. (2012) [55]	F4-rTMS sham	Ⓟ Ⓡ Ⓢ	15 16	47.2 ± 11.3 50.7 ± 8.6	20.0 43.8	20 Hz, 110% MT, 1,560 pulses	1 day	Pre, Within 3 days of stimulation	1 1	OCDS–5 items
Nakamura-Palacios et al.(2012) [46]	a-F3-tDCS sham	Ⓒ Ⓡ Ⓢ	49 49	48.8 ± 8.9 48.8 ± 8.9	8.2 8.2	1 mA, 10 min	1 day	Pre, Post	1 1	OCDS–5 items
Herremans et al. (2013) [62]	F4-rTMS sham	Ⓒ Ⓡ Ⓢ	29 29	48.1 ± 9.3 48.1 ± 9.3	34.5 34.5	20 Hz, 110% MT, 1,560 pulses	1 day	Pre, Post	1 1	OCDS–14 items
Klauss et al. (2014) [63]	a-F4 + c-F3 tDCS sham	Ⓟ Ⓡ Ⓓ	16 17	44.0 ± 7.8 45.5 ± 8.9	0.0 5.9	2 mA, 13 min	5 days	Pre, Post	10 10	OCDS–5 items
Ceccanti et al. (2015) [60]	mPFC-dTMS sham	Ⓟ Ⓡ Ⓓ	9 9	43.2 ± 11.1 47.3 ± 11.5	0.0 0.0	20 Hz, 120% MT, 1,500 pulses	2 weeks	Pre, 8 weeks follow up	10 10	VAS
den Uyl et al. (2015) [64]	a-F3-tDCS a-right IFG-tDCS sham	Ⓟ Ⓡ Ⓓ	14 15 12	21.1 ± 2.9 21.6 ± 3.2 22.4 ± 2.7	57.1 66.7 66.7	1 mA, 10 min	1 day	Pre, Post	1 1 1	AAAQ-subscale inclined
Herremans et al. (2015) [65]	F4-rTMS sham	Ⓟ Ⓡ Ⓓ	13 13	46.7 ± 10.4 43.7 ± 8.1	30.7 38.5	20 Hz, 110% MT, 1,560 pulses	1 day	Pre, Post	1 1	TLS craving score
Wietschorke et al. (2016) [66]	a-F4 + c-F3 tDCS sham	Ⓟ Ⓡ Ⓓ	15 15	42.6 ± 8.0 48.5 ± 9.7	46.7 26.7	1 mA, 20 min	1 day	Pre, Post	1 1	VAS–desire subscale
Addolorato et al. (2017) [67]	dlPFC-dTMS sham	Ⓟ Ⓡ Ⓓ	5 6	48.6 ± 9.9 48.6 ± 9.9	20.0 16.7	10 Hz, 100% MT, 1,000 pulses	4 weeks	Pre, Post	12 12	OCDS
Hanlon et al. (2017) [68]	FP1-cTBS sham	Ⓒ Ⓡ Ⓢ	24 24	27.0 ± 5.7 27.0 ± 5.7	29.1 29.1	5 Hz, 110% MT, 3,600 pulses	1 day	Pre, Post	1 1	Self-reported craving
den Uyl et al. (2018) [69]	a-F3-tDCS sham	Ⓕ Ⓡ Ⓓ	20 22	48.7 ± 7.3 48.2 ± 19.3	20.0 22.7	2 mA, 20 min	1 week	Pre, Within 1 week of last stimulation	4 4	PACS
Klauss et al. (2018) [70]	a-F4 + c-F3 tDCS sham	Ⓟ Ⓡ Ⓓ	23 22	46.3 ± 12.0 43.5 ± 10.2	21.7 13.6	2 mA, 20 min	5 weeks	Pre, Post	10 10	OCDS–5 items
Holla et al. (2020) [71]	a-F4 + c-F3 tDCS sham	Ⓟ Ⓡ Ⓓ	11 10	38.6 ± 7.1 39.4 ± 7.9	0 0	2 mA, 20 min	5 days	Pre, Post	5 5	ACQ-SF-R
Perini et al. (2020) [72]	IC-rTMS sham	Ⓟ Ⓡ Ⓓ	23 22	50.6 ± 10.4 53.5 ± 7.5	17.4 18.2	10 Hz, 120% MT, 1,500 pulses	3 weeks	Pre, 12 weeks follow up	15 15	PACS
Vanderhasselt et al.(2020) [73]	a-F4 + c-F3 tDCS sham	Ⓒ Ⓡ Ⓓ	45 45	21.2 ± 1.4 21.2 ± 1.4	33.0 33.0	2 mA, 20 min	1 day	Pre, Post	1 1	AAAQ-subscale inclined
Harel et al. (2022) [74]	mPFC-dTMS sham	Ⓟ Ⓡ Ⓓ	23 23	43.7 ± 8.7 42.5 ± 9.8	34.8 34.8	10 Hz, 100% MT, 3,000 pulses	3 weeks	Pre, 12 weeks follow up	15 15	PACS
Padula et al. (2024) [75]	F3-iTBS sham	Ⓟ Ⓡ Ⓓ	8 9	39.8 ± 9.8 48.5 ± 12.4	0 11.1	three pulse burst, 50 Hz, 100–110% MT, 10,332 pulses	10 days	Pre, Post	20 20	OCDS
Patil et al. (2024) [76]	a-F4 + c-F3 tDCS sham	Ⓟ Ⓡ Ⓢ	38 38	37.6 ± 8.5 36.6 ± 8.9	- -	2 mA, 20 min	5 days	Pre, Post	10 10	ACQ-NOW

Abbreviations: left dlPFC, F3; right dlPFC, F4; inferior frontal gyrus, IFG; medial prefrontal cortex, mPFC; left frontal pole, FP1; insular cortex, IC; anode, a; cathode, c; Parallel design, Ⓟ; Cross-over design, Ⓒ; Factorial design, Ⓕ; Randomized controlled trial, Ⓡ; Double-blind, Ⓓ; Single-blind, Ⓢ; Alcohol Urge Questionnaire, AUQ; Alcohol Craving Questionnaire, ACQ; Alcohol Craving Questionnaire-Short Form Revised, ACQ-SF-R; Obsessive Compulsive Drinking Scale, OCDS; Approach and Avoidance of Alcohol Questionnaire, AAAQ; Ten-point Likert Scales, TLS; Visual Analog Scale, VAS; Penn Alcohol Craving Scale, PACS

In this meta-analysis, NIBS was categorized into tDCS and TMS groups. The tDCS group included eight units of bilateral tDCS and four units of unilateral tDCS, while the TMS group consisted of five units of rTMS, three units of deep TMS (dTMS), one unit of continuous TBS (cTBS), and one unit of intermittent TBS (iTBS).

There were a total of 10 types of NIBS techniques, differentiated by site of stimulation and method of application, excluding sham controls. The specific types of NIBS used in this study are listed in Appendix Table 7. The most frequently applied NIBS technique was tDCS with the anode was positioned over the right dlPFC and the cathode over the left dlPFC (a-F4 + c-F3 tDCS), used in seven units, followed by rTMS over the right dlPFC (F4-rTMS), applied in four units.

In terms of the number of stimulation sessions, 11 units applied only a single session, representing half of all units. For tDCS group, seven out of 12 units applied a single session, whereas for TMS group, four out of 10 units applied a single session.

Participant characteristics revealed an average age range from 21.1 to 53.5 years. The percentage of female participants varied from 0 to 66.7%, although female participation was generally low across studies. Regarding alcohol consumption characteristics, many studies have not reported detailed results.

### Quality assessment

In terms of overall risk of bias, seven studies (35.0%) out of a total of 20 were classified as high risk, seven studies (35.0%) as unclear risk and six studies (30.0%) as low risk (Appendix Table 8). Notably, the allocation concealment domain (D2) was marked as unclear in 12 studies (60.0%), indicating a potential risk of selection bias. In terms of detection bias, seven studies (35.0%) were classified as having a high risk in the blinding of outcome assessment domain (D4), and 10 studies (50.0%) had an unclear risk of bias in the blinding of performance and personnel domain (D3). The certainty assessment of the quality of the evidence was moderate for change in craving severity and low for rate of adverse events (Appendix Table 9).

### Changes in craving severity

**Fig. 2:**
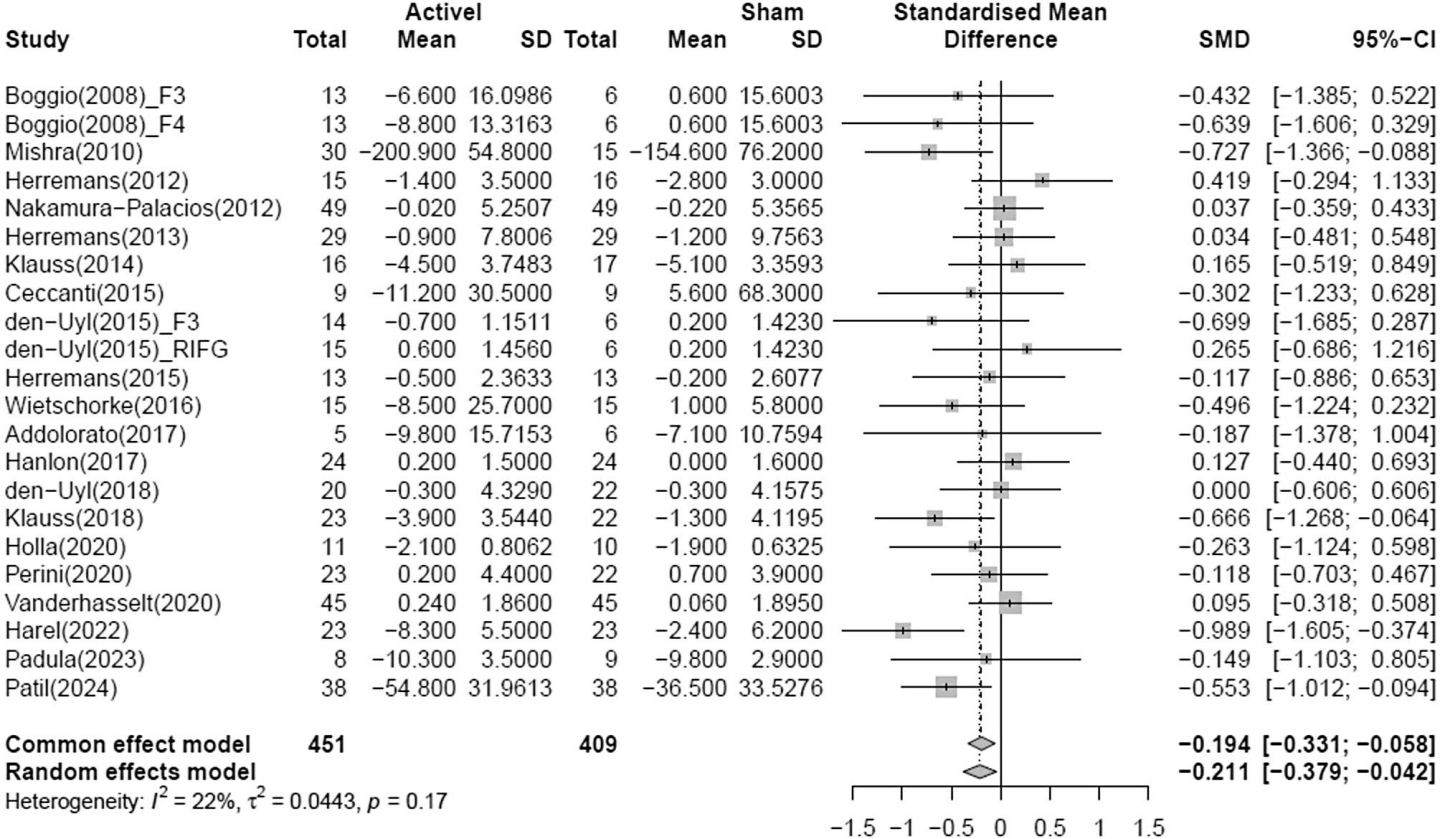
Forest plot of pooled effect estimates for reduction in alcohol severity in NIBS. NIBS, Non-invasive brain stimulation

A meta-analysis using the RE model to compare changes in craving severity between the NIBS and sham groups, demonstrated that the NIBS group significantly reduced craving severity compared to the sham group (SMD = -0.211; 95% CI = -0.379 to -0.042). Heterogeneity was low, with an I^2^ of 22.4% (*p* = 0.17) (Fig. 2).

Subgroup analyses were performed on the variables of on types of NIBS, stimulation parameters, time point of craving assessment, study design and quality assessment to identify moderators that influenced the reduction in craving severity. Subgroup analyses were conducted on a total of six variables (Table 2).

**Table 2:** Subgroup analysis of NIBS variables affecting reduction in alcohol craving severity

Comparison	Number of units	SMD (95% Cl)	I^2^	
Types of NIBS				
tDCS	12	-0.214 (-0.427 to -0.002)^*^	15.4%	
TMS	10	-0.202 (-0.489 to 0.086)	36.1%	
Site of stimulation				
dlPFC	17	-0.200 (-0.381 to -0.019)^*^	13.0%	
Other sites	5	-0.229 (-0.687 to 0.230)	53.4%	
Number of sessions				
Single-session	11	-0.020 (-0.206 to 0.166)	0.0%	
Multiple sessions	11	-0.388 (-0.620 to -0.156)^*^	12.5%	
Time point of craving assessment				
After stimulation	18	-0.126 (-0.291 to 0.038)	0.0%	
Follow up for at least 4 weeks	4	-0.553 (-0.979 to -0.126)^*^	35.1%	
Study design				
Parallel or factorial design	16	-0.311 (-0.520 to -0.101)^*^	19.6%	
Cross-over design	6	0.007 (-0.209 to 0.224)	0.0%	
Quality assessment				
Low risk of bias	6	-0.160 (-0.450 to 0.129)	0.0%	
Unclear or high risk of bias	16	-0.233 (-0.443 to -0.023)^*^	32.6%	

* P-value < 0.05

Among the types of NIBS, only the tDCS group (SMD = -0.214; 95% CI = -0.427 to -0.002) showed a significant reduction in alcohol craving severity compared to the sham group. Heterogeneity was low, with an I^2^ of 15.4% (*p* = 0.29). In relation to site of stimulation, a significant reduction in alcohol craving severity was observed only in the group where NIBS was applied to the dlPFC (SMD = -0.200; 95% CI = -0.381 to -0.019). Regarding the number of sessions, a significant reduction in alcohol craving severity was only found in the multi-session group (SMD = -0.388; 95% CI = -0.620 to -0.156). As for the time point of craving assessment, a significant reduction in alcohol craving severity was observed only in the group that underwent follow-up for at least 4 weeks after the last stimulation (SMD = -0.533; 95% CI = -0.979 to -0.126). In terms of study design, significant reduction in craving severity was observed in the group that used a parallel or factorial design (SMD = -0.311; 95% CI = -0.520 to -0.101). In terms of quality assessment, significant reduction was found in the group with unclear or high risk of bias (SMD = -0.233; 95% CI = -0.443 to -0.023).

### Safety (rate of adverse events)

Nine of the 22 units reported the occurrence of adverse events following NIBS or sham stimulation, with a total of 88 adverse events across five categories. The most common adverse event was headache, accounting for 44 reports (50.0%). The second most common adverse event was discomfort at the stimulation site (including scalp itching and tingling), with 37 reports (42.0%).

In the NIBS group, 28 cases of headache, 22 cases of discomfort (including scalp itching and tingling), and 4 cases of mood changes (such as anxiety) were reported as adverse events. In the sham group, there were 16 cases of headache, 15 cases of discomfort (including scalp itching and tingling), 1 case of pain, 1 case of mood changes (such as anxiety), and 1 case of seizure.

**Fig. 3:**
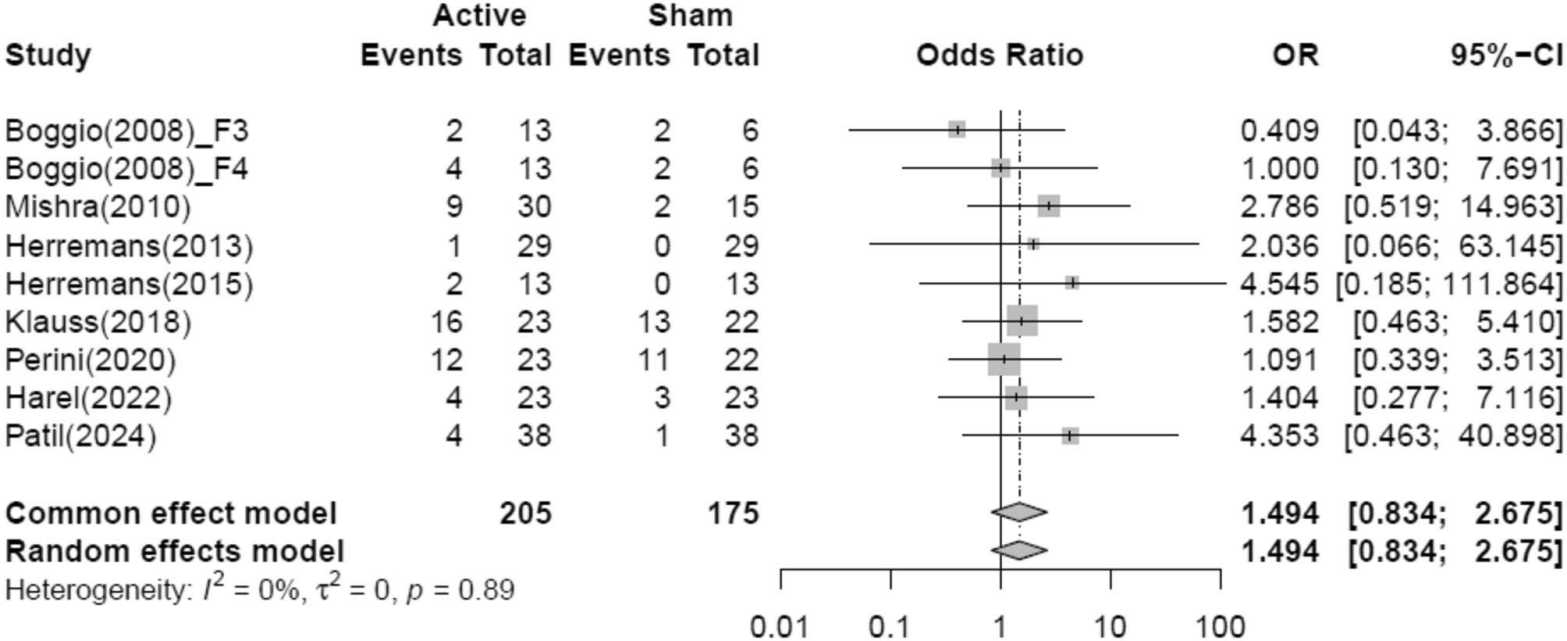
Forest plot of pooled effect estimates for safety (rate of adverse events) in NIBS. NIBS, Non-invasive brain stimulation

A meta-analysis using the RE model to compare the rate of adverse events between the NIBS and sham groups revealed that the NIBS group had a higher rate of adverse events than the sham group, though this difference was not significant (OR = 1.494; 95% CI = 0.834 to 2.675). Heterogeneity was very low, with an I^2^ of 0.0% (*p* = 0.89) (Fig. 3).

### Publication bias

**Fig. 4:**
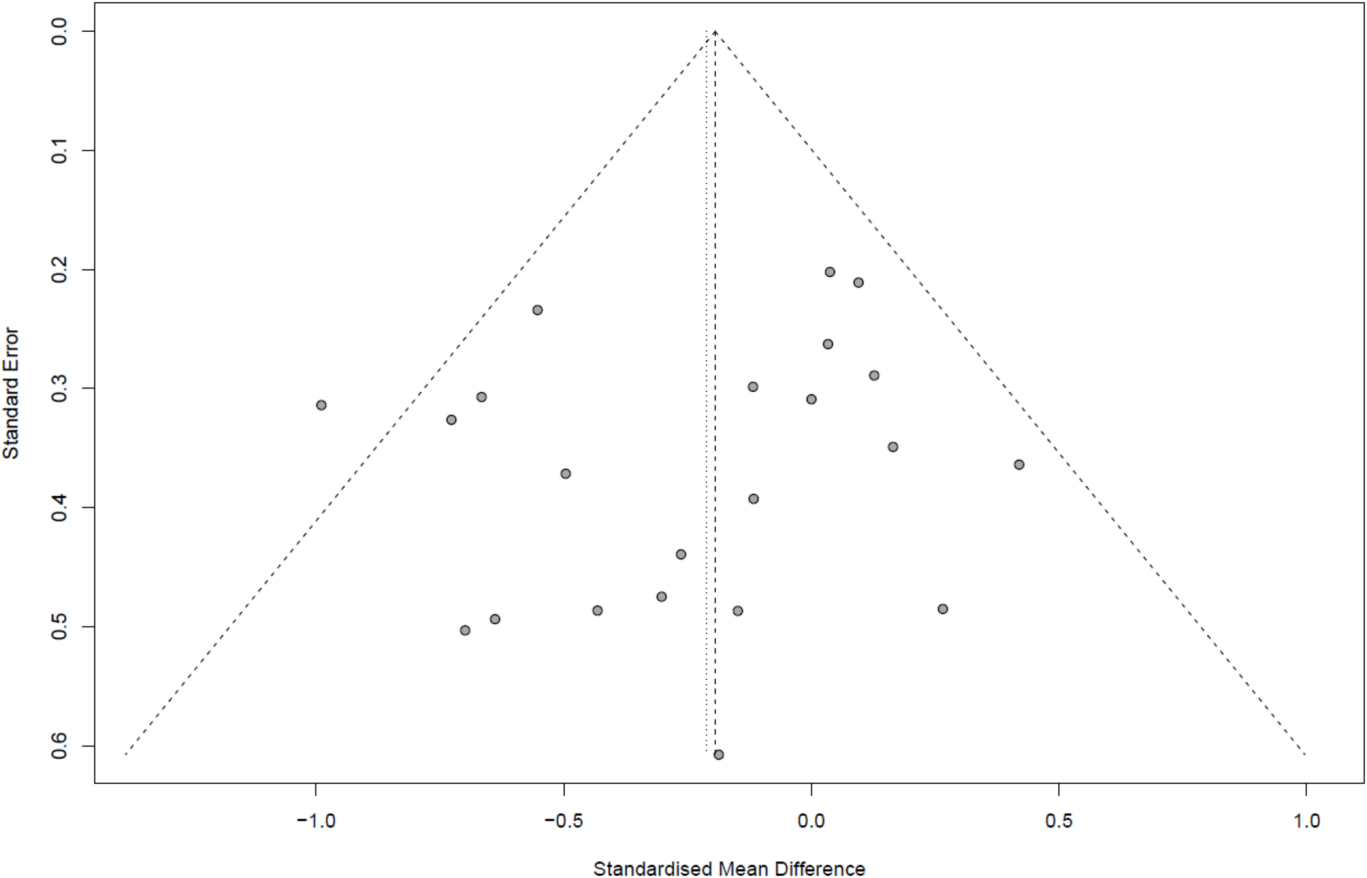
Funnel plot to assess publication bias among the included studies

To assess publication bias, we visually inspected the distribution of effect sizes using a funnel plot to detect any asymmetry among the studies. The results indicated that, aside from one study on the left side of the funnel, the studies were evenly distributed around the center line, suggesting no substantial risk of publication bias (Fig. 4). Additionally, Egger’s test was conducted to statistically assess the risk of publication bias, and the results showed no significant evidence of bias (*p* = 0.357).

## Discussion

This systematic review and meta-analysis assessed the efficacy of NIBS in reducing alcohol craving among patients with AUD, synthesizing data from 20 RCTs involving a total of 860 participants. A total of 22 units were included in the meta-analysis, with 12 units representing tDCS group and 10 units representing TMS group.

NIBS was significantly effective in reducing alcohol craving and the main adverse events were mild, including headache and discomfort at the stimulation site. Although these adverse events can be unpleasant, they were generally transient and not serious. One serious adverse event, a seizure, was reported only in the sham group and was not associated with the sham device. The meta-analysis revealed that although the rate of adverse events was higher in the NIBS group compared to the sham group, this difference was not significant. Therefore, NIBS appears to be a viable therapeutic option for reducing craving in patients with AUD. A comprehensive review of its efficacy and safety suggests positive treatment outcomes with manageable adverse events.

Subgroup analysis by NIBS type indicated that only the tDCS group showed a significant reduction in alcohol craving severity compared to the sham group. The TMS group did not show a significant reduction in craving severity compared to the sham group. Although there is still no clear conclusion about the appropriate site for stimulation, some studies have suggested that rTMS or cTBS primarily affects dopamine release when applied to the left dlPFC [47, 48]. In our study, there was only one unit in the TMS group that was applied to the left dlPFC. In addition, four of the 10 units applied only a single-session, and none of these cases showed a significant craving reduction effect, suggesting that this may also have influenced the results.

Application of NIBS to the dlPFC is known to regulate altered activity in the mesolimbic pathway, leading to a reduction in cravings [13, 49]. Recent studies in drug addiction suggest that the dlPFC plays a role in inhibitory control and, given its proximity to the scalp, has been proposed as an important neural target for NIBS [50–52]. Subgroup analyses of our study showed that NIBS significantly reduced craving when applied to the dlPFC.

Regarding the number of sessions, the single-session NIBS group did not show a significant reduction in craving, whereas the multi-session group showed a significant reduction. A meta-analysis evaluating the craving reduction effects of NIBS applied to the dlPFC also reported that multiple sessions are more effective than a single-session [53]. A single session of NIBS induces transient changes in cortical excitability, resulting in temporary reductions in craving. In contrast, multiple sessions of NIBS have demonstrated sustained effects, attributed to cumulative long-term potentiation (LTP) [53–56]. Therefore, it is considered appropriate to apply multiple sessions of NIBS rather than a single-session to reduce craving in patients with AUD.

NIBS can produce immediate effects that can be observed after stimulation, but its delayed effects can induce long-term neuroplasticity, allowing the stimulation effects to persist for hours to weeks [57–59]. In this study, significant effects were only observed in the group where craving was measured through follow-up beyond 4 weeks after last stimulation. In the follow-up for at least 4 weeks group, the craving reduction effect lasted longer after stimulation, whereas in the sham group the placebo effect dissipated quickly, leading to a sharp increase in craving measures. In particular, with dTMS, craving reduction was effective for up to 2 months after stimulation, and a reduction in the average number of drinks per day remained effective for up to 3 months [60]. However, given that only four TMS studies were included in the group that evaluated changes in craving severity with a follow-up period of at least 4 weeks, the results of this analysis should be interpreted with caution.

In terms of quality assessment, only the unclear or high risk of bias group showed a significant reduction in craving. All studies included in the unclear or high risk of bias group were found to have inadequate allocation concealment or blinding, suggesting the possibility of an overestimation of the effect size. In addition, the low risk of bias group had relatively fewer studies, which may have reduced statistical power. Overall, as most data included in the meta-analysis was derived from studies with an unclear or high risk of bias, this factor should be carefully considered when evaluating the reliability of the findings. Regarding the quality of evidence, the risk of bias was assessed as serious for both outcomes, change in alcohol craving severity and the rate of adverse events. In terms of imprecision, the rate of adverse events was assessed as serious. In contrast, for the outcome of change in the severity of alcohol craving, the total sample size met the optimal information size criterion, and the 95% CI was narrow and did not cross the line of no effect [61]. Therefore, imprecision was considered not serious for this outcome. These factors influenced the overall certainty of the evidence.

This study has some limitations. First, caution is needed when interpreting the results, as most of the included studies had small sample sizes. Second, a single-session may have a transient effect, these effects are likely to vary depending on the intensity and duration of stimulation, as well as individual characteristics, and it may be difficult to generalize the results. Consequently, studies that applied a single-session may not be suitable for accurately observing the effect of NIBS on the severity of alcohol craving. A meta-analysis that includes only studies utilizing multi-session protocols is necessary for a more accurate evaluation of the effects of NIBS. Third, of the 22 units included in this study, 18 units (81.8%) reported only craving measurements taken immediately after the last stimulation, while four units assessed long-term effects beyond at least 4 weeks after the last stimulation. However, these four units only included TMS, which is a limitation in objectively assessing the long-term effects of overall NIBS. Fourth, almost half of the RCTs included in this study were found to have unclear or high risk of bias regarding allocation concealment and blinding, which affected the overall quality of the evidence.

In conclusion, NIBS significantly reduced craving in patients with AUD compared to sham, positioning it as a promising treatment option for managing craving in AUD. While the safety outcomes appeared less favorable for NIBS, the differences in adverse event rates were not significant. The certainty of the evidence for changes in the severity of alcohol craving was moderate, and the incidence of adverse events was low. Among the types of NIBS, only tDCS showed a significant reduction in craving. In this study, a significant reduction in craving severity was observed when NIBS was applied to the dlPFC and when multiple sessions were administered. In addition, a significant reduction in craving severity due to delayed effects was observed even 4 weeks after NIBS stimulation, but this finding should be interpreted with caution. Future research should focus on including only multi-session studies and conducting larger studies to enable a more objective evaluation of long-term effects. Furthermore, it is necessary to propose the most effective stimulation parameters for each type of NIBS to reduce the severity of alcohol craving.

## Electronic supplementary material

Below is the link to the electronic supplementary material.

Supplementary Material 1

## Data Availability

The data that support the conclusions of this study can be provided upon request.

## Abbreviations

AUD: Alcohol use disorder
CI: Confidence interval
cTBS: Continuous TBS
DLPFC: Dorsolateral prefrontal cortex
DSM-5: Diagnostic and Statistical Manual of Mental Disorders
HF-rTMS: High-frequency rTMS
IFG: Inferior frontal gyrus
iTBS: Intermittent TBS
LF-rTMS: Low-frequency rTMS
MD: Mean difference
NIBS: Non-invasive brain stimulation
OR: Odds ratio
PFC: Prefrontal cortex
RCTs: Randomized controlled trials
rTMS: Repetitive transcranial magnetic stimulation
SMD: Standardized mean difference
SUD: Substance use disorder
TBS: Theta burst stimulation
tDCS: Transcranial direct current stimulation
