# Supplementary material for: Efficacy of non-invasive brain stimulation in reducing craving in patients with alcohol use disorder: systematic review and meta-analysis
Source: BMC Psychiatry. 2025 May 16;25:496. doi: 10.1186/s12888-025-06883-4 (PMC12085020; doi:10.1186/s12888-025-06883-4)
Supplement: Supplementary file 1 — Supplementary Material 1 [file 12888_2025_6883_MOESM1_ESM.docx]

**Supplementary material**

The following supplementary material accompanies the article titled “*Efficacy of non-invasive brain stimulation in reducing craving in patients with alcohol use disorder : Systematic review and meta-analysis.”*

Table of contents

Appendix Table 1. PRISMA 2020 checklist 3

Appendix Table 2. PubMed database search strategy 6

Appendix Table 3. Embase database search strategy 9

Appendix Table 4. Cochrane Library database search strategy 13

Appendix Table 5. PsycINFO database search strategy 15

Appendix Table 6. Risk of bias assessment criteria 16

Appendix Table 7. Types of NIBS 17

Appendix Table 8. Assessment of the risk of bias for studies 18

Appendix Table 9. Assessment of quality of evidence 19

Appendix Table 1. PRISMA 2020 checklist

| **Section and Topic** | **Item #** | **Checklist item** | **Location where item is reported** |
| --- | --- | --- | --- |
| **TITLE** | | |  |
| Title | 1 | Identify the report as a systematic review. | 1 page |
| **ABSTRACT** | | |  |
| Abstract | 2 | See the PRISMA 2020 for Abstracts checklist. | 1-2 pages |
| **INTRODUCTION** | | |  |
| Rationale | 3 | Describe the rationale for the review in the context of existing knowledge. | 3-5 pages |
| Objectives | 4 | Provide an explicit statement of the objective(s) or question(s) the review addresses. | 5 page |
| **METHODS** | | |  |
| Eligibility criteria | 5 | Specify the inclusion and exclusion criteria for the review and how studies were grouped for the syntheses. | 5-6 pages |
| Information sources | 6 | Specify all databases, registers, websites, organisations, reference lists and other sources searched or consulted to identify studies. Specify the date when each source was last searched or consulted. | 5 page |
| Search strategy | 7 | Present the full search strategies for all databases, registers and websites, including any filters and limits used. | Appendix Tables 2-5 |
| Selection process | 8 | Specify the methods used to decide whether a study met the inclusion criteria of the review, including how many reviewers screened each record and each report retrieved, whether they worked independently, and if applicable, details of automation tools used in the process. | 5 page |
| Data collection process | 9 | Specify the methods used to collect data from reports, including how many reviewers collected data from each report, whether they worked independently, any processes for obtaining or confirming data from study investigators, and if applicable, details of automation tools used in the process. | 6 page |
| Data items | 10a | List and define all outcomes for which data were sought. Specify whether all results that were compatible with each outcome domain in each study were sought (e.g. for all measures, time points, analyses), and if not, the methods used to decide which results to collect. | 6 page |
|  | 10b | List and define all other variables for which data were sought (e.g. participant and intervention characteristics, funding sources). Describe any assumptions made about any missing or unclear information. | 6 page |
| Study risk of bias assessment | 11 | Specify the methods used to assess risk of bias in the included studies, including details of the tool(s) used, how many reviewers assessed each study and whether they worked independently, and if applicable, details of automation tools used in the process. | 6-7 pages |
| Effect measures | 12 | Specify for each outcome the effect measure(s) (e.g. risk ratio, mean difference) used in the synthesis or presentation of results. | 7 page |
| Synthesis methods | 13a | Describe the processes used to decide which studies were eligible for each synthesis (e.g. tabulating the study intervention characteristics and comparing against the planned groups for each synthesis (item #5)). | Table 1, Appendix Table 7 |
|  | 13b | Describe any methods required to prepare the data for presentation or synthesis, such as handling of missing summary statistics, or data conversions. | 7-8 pages |
|  | 13c | Describe any methods used to tabulate or visually display results of individual studies and syntheses. | 7-8 pages |
|  | 13d | Describe any methods used to synthesize results and provide a rationale for the choice(s). If meta-analysis was performed, describe the model(s), method(s) to identify the presence and extent of statistical heterogeneity, and software package(s) used. | 7-8 pages |
|  | 13e | Describe any methods used to explore possible causes of heterogeneity among study results (e.g. subgroup analysis, meta-regression). | 7-8 pages |
|  | 13f | Describe any sensitivity analyses conducted to assess robustness of the synthesized results. | 7-8 pages |
| Reporting bias assessment | 14 | Describe any methods used to assess risk of bias due to missing results in a synthesis (arising from reporting biases). | Appendix Table 6 |
| Certainty assessment | 15 | Describe any methods used to assess certainty (or confidence) in the body of evidence for an outcome. | 7-8 pages |
| **RESULTS** | | |  |
| Study selection | 16a | Describe the results of the search and selection process, from the number of records identified in the search to the number of studies included in the review, ideally using a flow diagram. | 9 page, Fig 1 |
|  | 16b | Cite studies that might appear to meet the inclusion criteria, but which were excluded, and explain why they were excluded. | 9 page, Fig 1 |
| Study characteristics | 17 | Cite each included study and present its characteristics. | 9 page |
| Risk of bias in studies | 18 | Present assessments of risk of bias for each included study. | 10 page, Appendix Table 8 |
| Results of individual studies | 19 | For all outcomes, present, for each study: (a) summary statistics for each group (where appropriate) and (b) an effect estimate and its precision (e.g. confidence/credible interval), ideally using structured tables or plots. | Fig 2-4 |
| Results of syntheses | 20a | For each synthesis, briefly summarise the characteristics and risk of bias among contributing studies. | 7 page |
|  | 20b | Present results of all statistical syntheses conducted. If meta-analysis was done, present for each the summary estimate and its precision (e.g. confidence/credible interval) and measures of statistical heterogeneity. If comparing groups, describe the direction of the effect. | 7-9 pages, Fig 2-4 |
|  | 20c | Present results of all investigations of possible causes of heterogeneity among study results. | 9-13 pages |
|  | 20d | Present results of all sensitivity analyses conducted to assess the robustness of the synthesized results. | 9-13 pages |
| Reporting biases | 21 | Present assessments of risk of bias due to missing results (arising from reporting biases) for each synthesis assessed. | Appendix Table 8 |
| Certainty of evidence | 22 | Present assessments of certainty (or confidence) in the body of evidence for each outcome assessed. | 9-13 pages |
| **DISCUSSION** | | |  |
| Discussion | 23a | Provide a general interpretation of the results in the context of other evidence. | 13-14 pages |
|  | 23b | Discuss any limitations of the evidence included in the review. | 16-17 pages |
|  | 23c | Discuss any limitations of the review processes used. | 16-17 pages |
|  | 23d | Discuss implications of the results for practice, policy, and future research. | 16-17 pages |
| **OTHER INFORMATION** | | |  |
| Registration and protocol | 24a | Provide registration information for the review, including register name and registration number, or state that the review was not registered. | 5 page |
|  | 24b | Indicate where the review protocol can be accessed, or state that a protocol was not prepared. | 5 page |
|  | 24c | Describe and explain any amendments to information provided at registration or in the protocol. | 5 page, refer to PROSPERO database  (CRD42024567484) |
| Support | 25 | Describe sources of financial or non-financial support for the review, and the role of the funders or sponsors in the review. | 19 page |
| Competing interests | 26 | Declare any competing interests of review authors. | 19 page |
| Availability of data, code and other materials | 27 | Report which of the following are publicly available and where they can be found: template data collection forms; data extracted from included studies; data used for all analyses; analytic code; any other materials used in the review. | 18 page |

Appendix Table 2. PubMed database search strategy

| **Search** | **Search terms** |
| --- | --- |
| #1 | "Alcoholism"[Mesh] |
| #2 | "Alcoholism"[TW] OR "Alcohol Dependence"[TW] OR "Dependence, Alcohol"[TW] OR "Alcohol Addiction"[TW] OR "Addiction, Alcohol"[TW] OR "Alcoholic Intoxication, Chronic"[TW] OR "Chronic Alcoholic Intoxication"[TW] OR "Intoxication, Chronic Alcoholic"[TW] OR "Alcohol Use Disorder"[TW] OR "Alcohol Use Disorders"[TW] OR "Use Disorder, Alcohol"[TW] OR "Use Disorders, Alcohol"[TW] OR "Alcohol Abuse"[TW] OR "Abuse, Alcohol"[TW] |
| #3 | "Substance-Related Disorders"[Mesh] |
| #4 | "Substance-Related Disorders"[TW] OR "Drug Abuse"[TW] OR "Abuse, Drug"[TW] OR "Drug Dependence"[TW] OR "Dependence, Drug"[TW] OR "Drug Addiction"[TW] OR "Addiction, Drug"[TW] OR "Substance Use Disorders"[TW] OR "Disorder, Substance Use"[TW] OR "Substance Use Disorder"[TW] OR "Drug Use Disorders"[TW] OR "Disorder, Drug Use"[TW] OR "Drug Use Disorder"[TW] OR "Organic Mental Disorders, Substance-Induced"[TW] OR "Organic Mental Disorders, Substance Induced"[TW] OR "Substance Abuse"[TW] OR "Abuse, Substance"[TW] OR "Abuses, Substance"[TW] OR "Substance Abuses"[TW] OR "Substance Dependence"[TW] OR "Dependence, Substance"[TW] OR "Substance Addiction"[TW] OR "Addiction, Substance"[TW] OR "Prescription Drug Abuse"[TW] OR "Abuse, Prescription Drug"[TW] OR "Drug Abuse, Prescription"[TW] OR "Drug Habituation"[TW] OR "Habituation, Drug"[TW] |
| #5 | "Alcoholics"[Mesh] |
| #6 | "Alcoholics"[TW] OR "Alcoholic"[TW] OR "Skid Row Alcoholics"[TW] OR "Alcoholic, Skid Row"[TW] OR "Alcoholics, Skid Row"[TW] OR "Skid Row Alcoholic"[TW] |
| #7 | "Alcohol-Related Disorders"[Mesh] |
| #8 | "Alcohol-Related Disorders"[TW] OR "Alcohol Related Disorders"[TW] OR "Alcohol-Related Disorder"[TW] OR "Disorder, Alcohol-Related"[TW] OR "Disorders, Alcohol-Rel"[TW] |
| #9  Combine | ((((((("Alcoholism"[Mesh]) OR ("Alcoholism"[TW] OR "Alcohol Dependence"[TW] OR "Dependence, Alcohol"[TW] OR "Alcohol Addiction"[TW] OR "Addiction, Alcohol"[TW] OR "Alcoholic Intoxication, Chronic"[TW] OR "Chronic Alcoholic Intoxication"[TW] OR "Intoxication, Chronic Alcoholic"[TW] OR "Alcohol Use Disorder"[TW] OR "Alcohol Use Disorders"[TW] OR "Use Disorder, Alcohol"[TW] OR "Use Disorders, Alcohol"[TW] OR "Alcohol Abuse"[TW] OR "Abuse, Alcohol"[TW])) OR ("Substance-Related Disorders"[Mesh])) OR ("Substance-Related Disorders"[TW] OR "Drug Abuse"[TW] OR "Abuse, Drug"[TW] OR "Drug Dependence"[TW] OR "Dependence, Drug"[TW] OR "Drug Addiction"[TW] OR "Addiction, Drug"[TW] OR "Substance Use Disorders"[TW] OR "Disorder, Substance Use"[TW] OR "Substance Use Disorder"[TW] OR "Drug Use Disorders"[TW] OR "Disorder, Drug Use"[TW] OR "Drug Use Disorder"[TW] OR "Organic Mental Disorders, Substance-Induced"[TW] OR "Organic Mental Disorders, Substance Induced"[TW] OR "Substance Abuse"[TW] OR "Abuse, Substance"[TW] OR "Abuses, Substance"[TW] OR "Substance Abuses"[TW] OR "Substance Dependence"[TW] OR "Dependence, Substance"[TW] OR "Substance Addiction"[TW] OR "Addiction, Substance"[TW] OR "Prescription Drug Abuse"[TW] OR "Abuse, Prescription Drug"[TW] OR "Drug Abuse, Prescription"[TW] OR "Drug Habituation"[TW] OR "Habituation, Drug"[TW])) OR ("Alcoholics"[Mesh])) OR ("Alcoholics"[TW] OR "Alcoholic"[TW] OR "Skid Row Alcoholics"[TW] OR "Alcoholic, Skid Row"[TW] OR "Alcoholics, Skid Row"[TW] OR "Skid Row Alcoholic"[TW])) OR ("Alcohol-Related Disorders"[Mesh])) OR ("Alcohol-Related Disorders"[TW] OR "Alcohol Related Disorders"[TW] OR "Alcohol-Related Disorder"[TW] OR "Disorder, Alcohol-Related"[TW] OR "Disorders, Alcohol-Rel"[TW]) |
| #10 | "non-invasive brain stimulation"[TW] OR "NIBS"[TW] OR "Non-invasive neurostimulation"[TW] OR "Neuro stimulation"[TW] OR "Cortical stimulation"[TW] |
| #11 | "Deep Brain Stimulation"[Mesh] |
| #12 | "Deep Brain Stimulation"[TW] OR "Brain Stimulations, Deep"[TW] OR "Deep Brain Stimulations"[TW] OR "Stimulation, Deep Brain"[TW] OR "Stimulations, Deep Brain"[TW] OR "Brain Stimulation, Deep"[TW] OR "Electrical Stimulation of the Brain"[TW] OR "Electrical brain stimulation"[TW] OR "EBS"[TW] OR "Brain stimulation"[TW] |
| #13 | "Transcranial Magnetic Stimulation"[Mesh] |
| #14 | "Transcranial Magnetic Stimulation"[TW] OR "Magnetic Stimulation, Transcranial"[TW] OR "Magnetic Stimulations, Transcranial"[TW] OR "Stimulation, Transcranial Magnetic"[TW] OR "Stimulations, Transcranial Magnetic"[TW] OR "Transcranial Magnetic Stimulations"[TW] OR "Transcranial Magnetic Stimulation, Single Pulse"[TW] OR "Transcranial Magnetic Stimulation, Paired Pulse"[TW] OR "Transcranial Magnetic Stimulation, RepetitiveTMS"[TW] OR "repetitive transcranial magnetic stimulation"[TW] OR "rTMS"[TW] OR "TMS"[TW] OR "repetitive transcranial magnetic stimulation"[TW] OR "rTMS"[TW] |
| #15 | "Accelerated transcranial magnetic stimulation"[TW] OR "aTMS"[TW] OR "Priming transcranial magnetic stimulation"[TW] OR "pTMS"[TW] OR "Deep transcranial magnetic stimulation"[TW] OR "dTMS"[TW] OR "Theta burst stimulation"[TW] OR "TBS"[TW] OR "Synchronised transcranial magnetic stimulation"[TW] OR "sTMS"[TW] |
| #16 | "Transcranial Direct Current Stimulation"[Mesh] |
| #17 | "Transcranial Direct Current Stimulation"[TW] OR "tDCS"[TW] OR "Cathodal Stimulation Transcranial Direct Current Stimulation"[TW] OR "Cathodal Stimulation tDCS"[TW] OR "Cathodal Stimulation tDCSs"[TW] OR "Stimulation tDCS, Cathodal"[TW] OR "Stimulation tDCSs, Cathodal"[TW] OR "tDCS, Cathodal Stimulation"[TW] OR "tDCSs, Cathodal Stimulation"[TW] OR "Transcranial Random Noise Stimulation"[TW] OR "Transcranial Alternating Current Stimulation"[TW] OR "Transcranial Electrical Stimulation"[TW] OR "Electrical Stimulation, Transcranial"[TW] OR "Electrical Stimulations, Transcranial"[TW] OR "Stimulation, Transcranial Electrical"[TW] OR "Stimulations, Transcranial Electrical"[TW] OR "Transcranial Electrical Stimulations"[TW] OR "Anodal Stimulation Transcranial Direct Current Stimulation"[TW] OR "Anodal Stimulation tDCS"[TW] OR "Anodal Stimulation tDCSs"[TW] OR "Stimulation tDCS, Anodal"[TW] OR "Stimulation tDCSs, Anodal"[TW] OR "tDCS, Anodal Stimulation"[TW] OR "tDCSs, Anodal Stimulation"[TW] OR "Repetitive Transcranial Electrical Stimulation"[TW] OR "tACS"[TW] |
| #18 | ((((((("non-invasive brain stimulation"[TW] OR "NIBS"[TW] OR "Non-invasive neurostimulation"[TW] OR "Neuro stimulation"[TW] OR "Cortical stimulation"[TW]) OR ("Deep Brain Stimulation"[Mesh])) OR ("Deep Brain Stimulation"[TW] OR "Brain Stimulations, Deep"[TW] OR "Deep Brain Stimulations"[TW] OR "Stimulation, Deep Brain"[TW] OR "Stimulations, Deep Brain"[TW] OR "Brain Stimulation, Deep"[TW] OR "Electrical Stimulation of the Brain"[TW] OR "Electrical brain stimulation"[TW] OR "EBS"[TW] OR "Brain stimulation"[TW)) OR ("Transcranial Magnetic Stimulation"[Mesh])) OR ("Transcranial Magnetic Stimulation"[TW] OR "Magnetic Stimulation, Transcranial"[TW] OR "Magnetic Stimulations, Transcranial"[TW] OR "Stimulation, Transcranial Magnetic"[TW] OR "Stimulations, Transcranial Magnetic"[TW] OR "Transcranial Magnetic Stimulations"[TW] OR "Transcranial Magnetic Stimulation, Single Pulse"[TW] OR "Transcranial Magnetic Stimulation, Paired Pulse"[TW] OR "Transcranial Magnetic Stimulation, RepetitiveTMS"[TW] OR "repetitive transcranial magnetic stimulation"[TW] OR "rTMS"[TW] OR "TMS"[TW] OR "repetitive transcranial magnetic stimulation"[TW] OR "rTMS"[TW])) OR ("Accelerated transcranial magnetic stimulation"[TW] OR "aTMS"[TW] OR "Priming transcranial magnetic stimulation"[TW] OR "pTMS"[TW] OR "Deep transcranial magnetic stimulation"[TW] OR "dTMS"[TW] OR "Theta burst stimulation"[TW] OR "TBS"[TW] OR "Synchronised transcranial magnetic stimulation"[TW] OR "sTMS"[TW])) OR ("Transcranial Direct Current Stimulation"[Mesh])) OR ("Transcranial Direct Current Stimulation"[TW] OR "tDCS"[TW] OR "Cathodal Stimulation Transcranial Direct Current Stimulation"[TW] OR "Cathodal Stimulation tDCS"[TW] OR "Cathodal Stimulation tDCSs"[TW] OR "Stimulation tDCS, Cathodal"[TW] OR "Stimulation tDCSs, Cathodal"[TW] OR "tDCS, Cathodal Stimulation"[TW] OR "tDCSs, Cathodal Stimulation"[TW] OR "Transcranial Random Noise Stimulation"[TW] OR "Transcranial Alternating Current Stimulation"[TW] OR "Transcranial Electrical Stimulation"[TW] OR "Electrical Stimulation, Transcranial"[TW] OR "Electrical Stimulations, Transcranial"[TW] OR "Stimulation, Transcranial Electrical"[TW] OR "Stimulations, Transcranial Electrical"[TW] OR "Transcranial Electrical Stimulations"[TW] OR "Anodal Stimulation Transcranial Direct Current Stimulation"[TW] OR "Anodal Stimulation tDCS"[TW] OR "Anodal Stimulation tDCSs"[TW] OR "Stimulation tDCS, Anodal"[TW] OR "Stimulation tDCSs, Anodal"[TW] OR "tDCS, Anodal Stimulation"[TW] OR "tDCSs, Anodal Stimulation"[TW] OR "Repetitive Transcranial Electrical Stimulation"[TW] OR "tACS"[TW]) |
| #19  Combine | #9 AND #18 |

Appendix Table 3. EMBASE database search strategy

| **Search** | **Search terms** |
| --- | --- |
| #1 | "alcoholism"/exp |
| #2 | "Alcoholism":ti,ab,kw,de OR "Alcohol Dependence":ti,ab,kw,de OR "Dependence, Alcohol":ti,ab,kw,de OR "Alcohol Addiction":ti,ab,kw,de OR "Addiction, Alcohol":ti,ab,kw,de OR "Alcoholic Intoxication, Chronic":ti,ab,kw,de OR "Chronic Alcoholic Intoxication":ti,ab,kw,de OR "Intoxication, Chronic Alcoholic":ti,ab,kw,de OR "Alcohol Use Disorder":ti,ab,kw,de OR "Alcohol Use Disorders":ti,ab,kw,de OR "Use Disorder, Alcohol":ti,ab,kw,de OR "Use Disorders, Alcohol":ti,ab,kw,de OR "Alcohol Abuse":ti,ab,kw,de OR "Abuse, Alcohol":ti,ab,kw,de |
| #3 | "drug dependence"/exp |
| #4 | "Substance-Related Disorders":ti,ab,kw,de OR "Drug Abuse":ti,ab,kw,de OR "Abuse, Drug":ti,ab,kw,de OR "Drug Dependence":ti,ab,kw,de OR "Dependence, Drug":ti,ab,kw,de OR "Drug Addiction":ti,ab,kw,de OR "Addiction, Drug":ti,ab,kw,de OR "Substance Use Disorders":ti,ab,kw,de OR "Disorder, Substance Use":ti,ab,kw,de OR "Substance Use Disorder":ti,ab,kw,de OR "Drug Use Disorders":ti,ab,kw,de OR "Disorder, Drug Use":ti,ab,kw,de OR "Drug Use Disorder":ti,ab,kw,de OR "Organic Mental Disorders, Substance-Induced":ti,ab,kw,de OR "Organic Mental Disorders, Substance Induced":ti,ab,kw,de OR "Substance Abuse":ti,ab,kw,de OR "Abuse, Substance":ti,ab,kw,de OR "Abuses, Substance":ti,ab,kw,de OR "Substance Abuses":ti,ab,kw,de OR "Substance Dependence":ti,ab,kw,de OR "Dependence, Substance":ti,ab,kw,de OR "Substance Addiction":ti,ab,kw,de OR "Addiction, Substance":ti,ab,kw,de OR "Prescription Drug Abuse":ti,ab,kw,de OR "Abuse, Prescription Drug":ti,ab,kw,de OR "Drug Abuse, Prescription":ti,ab,kw,de OR "Drug Habituation":ti,ab,kw,de OR "Habituation, Drug":ti,ab,kw,de OR "addict":ti,ab,kw,de OR "addiction, drug":ti,ab,kw,de OR "dependence, drug":ti,ab,kw,de OR "drug addict":ti,ab,kw,de OR "drug addiction":ti,ab,kw,de OR "drug dependence model":ti,ab,kw,de OR "drug dependency":ti,ab,kw,de OR "drug facilitation":ti,ab,kw,de OR "drug habituation":ti,ab,kw,de OR "drug physical dependence":ti,ab,kw,de OR "physical dependence":ti,ab,kw,de OR "substance addiction":ti,ab,kw,de OR "substance dependence":ti,ab,kw,de OR "substance dependency":ti,ab,kw,de OR "substance use disorder":ti,ab,kw,de OR "substance use disorders":ti,ab,kw,de OR "substance-related disorder":ti,ab,kw,de OR "substance-related disorders":ti,ab,kw,de OR "toxicomania":ti,ab,kw,de OR "toxicomanias":ti,ab,kw,de OR "toxicomanie":ti,ab,kw,de |
| #5 | "Alcoholics":ti,ab,kw,de OR "Alcoholic":ti,ab,kw,de OR "Skid Row Alcoholics":ti,ab,kw,de OR "Alcoholic, Skid Row":ti,ab,kw,de OR "Alcoholics, Skid Row":ti,ab,kw,de OR "Skid Row Alcoholic":ti,ab,kw,de |
| #6 | "Alcohol-Related Disorders":ti,ab,kw,de OR "Alcohol Related Disorders":ti,ab,kw,de OR "Alcohol-Related Disorder":ti,ab,kw,de OR "Disorder, Alcohol-Related":ti,ab,kw,de OR "Disorders, Alcohol-Rel":ti,ab,kw,de |
| #7  Combine | "alcoholism"/exp OR "Alcoholism":ti,ab,kw,de OR "Alcohol Dependence":ti,ab,kw,de OR "Dependence, Alcohol":ti,ab,kw,de OR "Alcohol Addiction":ti,ab,kw,de OR "Addiction, Alcohol":ti,ab,kw,de OR "Alcoholic Intoxication, Chronic":ti,ab,kw,de OR "Chronic Alcoholic Intoxication":ti,ab,kw,de OR "Intoxication, Chronic Alcoholic":ti,ab,kw,de OR "Alcohol Use Disorder":ti,ab,kw,de OR "Alcohol Use Disorders":ti,ab,kw,de OR "Use Disorder, Alcohol":ti,ab,kw,de OR "Use Disorders, Alcohol":ti,ab,kw,de OR "Alcohol Abuse":ti,ab,kw,de OR "Abuse, Alcohol":ti,ab,kw,de OR "drug dependence"/exp OR "Substance-Related Disorders":ti,ab,kw,de OR "Drug Abuse":ti,ab,kw,de OR "Abuse, Drug":ti,ab,kw,de OR "Drug Dependence":ti,ab,kw,de OR "Dependence, Drug":ti,ab,kw,de OR "Drug Addiction":ti,ab,kw,de OR "Addiction, Drug":ti,ab,kw,de OR "Substance Use Disorders":ti,ab,kw,de OR "Disorder, Substance Use":ti,ab,kw,de OR "Substance Use Disorder":ti,ab,kw,de OR "Drug Use Disorders":ti,ab,kw,de OR "Disorder, Drug Use":ti,ab,kw,de OR "Drug Use Disorder":ti,ab,kw,de OR "Organic Mental Disorders, Substance-Induced":ti,ab,kw,de OR "Organic Mental Disorders, Substance Induced":ti,ab,kw,de OR "Substance Abuse":ti,ab,kw,de OR "Abuse, Substance":ti,ab,kw,de OR "Abuses, Substance":ti,ab,kw,de OR "Substance Abuses":ti,ab,kw,de OR "Substance Dependence":ti,ab,kw,de OR "Dependence, Substance":ti,ab,kw,de OR "Substance Addiction":ti,ab,kw,de OR "Addiction, Substance":ti,ab,kw,de OR "Prescription Drug Abuse":ti,ab,kw,de OR "Abuse, Prescription Drug":ti,ab,kw,de OR "Drug Abuse, Prescription":ti,ab,kw,de OR "Drug Habituation":ti,ab,kw,de OR "Habituation, Drug":ti,ab,kw,de OR "addict":ti,ab,kw,de OR "addiction, drug":ti,ab,kw,de OR "dependence, drug":ti,ab,kw,de OR "drug addict":ti,ab,kw,de OR "drug addiction":ti,ab,kw,de OR "drug dependence model":ti,ab,kw,de OR "drug dependency":ti,ab,kw,de OR "drug facilitation":ti,ab,kw,de OR "drug habituation":ti,ab,kw,de OR "drug physical dependence":ti,ab,kw,de OR "physical dependence":ti,ab,kw,de OR "substance addiction":ti,ab,kw,de OR "substance dependence":ti,ab,kw,de OR "substance dependency":ti,ab,kw,de OR "substance use disorder":ti,ab,kw,de OR "substance use disorders":ti,ab,kw,de OR "substance-related disorder":ti,ab,kw,de OR "substance-related disorders":ti,ab,kw,de OR "toxicomania":ti,ab,kw,de OR "toxicomanias":ti,ab,kw,de OR "toxicomanie":ti,ab,kw,de OR "Alcoholics":ti,ab,kw,de OR "Alcoholic":ti,ab,kw,de OR "Skid Row Alcoholics":ti,ab,kw,de OR "Alcoholic, Skid Row":ti,ab,kw,de OR "Alcoholics, Skid Row":ti,ab,kw,de OR "Skid Row Alcoholic":ti,ab,kw,de OR "Alcohol-Related Disorders":ti,ab,kw,de OR "Alcohol Related Disorders":ti,ab,kw,de OR "Alcohol-Related Disorder":ti,ab,kw,de OR "Disorder, Alcohol-Related":ti,ab,kw,de OR "Disorders, Alcohol-Rel":ti,ab,kw,de |
| #8 | "non-invasive brain stimulation":ti,ab,kw,de OR "NIBS":ti,ab,kw,de OR "Non-invasive neurostimulation":ti,ab,kw,de OR "Neuro stimulation":ti,ab,kw,de OR "Cortical stimulation":ti,ab,kw,de |
| #9 | "brain depth stimulation"/exp |
| #10 | "deep brain stimulator"/exp |
| #11 | "Deep Brain Stimulation":ti,ab,kw,de OR "Brain Stimulations, Deep":ti,ab,kw,de OR "Deep Brain Stimulations":ti,ab,kw,de OR "Stimulation, Deep Brain":ti,ab,kw,de OR "Stimulations, Deep Brain":ti,ab,kw,de OR "Brain Stimulation, Deep":ti,ab,kw,de OR "Electrical Stimulation of the Brain":ti,ab,kw,de OR "Electrical brain stimulation":ti,ab,kw,de OR "EBS":ti,ab,kw,de OR "Brain stimulation":ti,ab,kw,de OR "brain excitation":ti,ab,kw,de OR "brain stimulation":ti,ab,kw,de OR "brain stimulus":ti,ab,kw,de OR "deep brain stimulation":ti,ab,kw,de OR "excitation, brain":ti,ab,kw,de OR "deep brain electrical stimulation system":ti,ab,kw,de OR "deep brain stimulation device":ti,ab,kw,de OR "deep brain stimulation system":ti,ab,kw,de |
| #12 | "transcranial magnetic stimulation"/exp |
| #13 | "Transcranial Magnetic Stimulation":ti,ab,kw,de OR "Magnetic Stimulation, Transcranial":ti,ab,kw,de OR "Magnetic Stimulations, Transcranial":ti,ab,kw,de OR "Stimulation, Transcranial Magnetic":ti,ab,kw,de OR "Stimulations, Transcranial Magnetic":ti,ab,kw,de OR "Transcranial Magnetic Stimulations":ti,ab,kw,de OR "Transcranial Magnetic Stimulation, Single Pulse":ti,ab,kw,de OR "Transcranial Magnetic Stimulation, Paired Pulse":ti,ab,kw,de OR "Transcranial Magnetic Stimulation, RepetitiveTMS":ti,ab,kw,de OR "repetitive transcranial magnetic stimulation":ti,ab,kw,de OR "rTMS":ti,ab,kw,de OR "TMS":ti,ab,kw,de OR "repetitive transcranial magnetic stimulation":ti,ab,kw,de OR "rTMS":ti,ab,kw,de |
| #14 | "Accelerated transcranial magnetic stimulation":ti,ab,kw,de OR "aTMS":ti,ab,kw,de OR "Priming transcranial magnetic stimulation":ti,ab,kw,de OR "pTMS":ti,ab,kw,de OR "Deep transcranial magnetic stimulation":ti,ab,kw,de OR "dTMS":ti,ab,kw,de OR "Theta burst stimulation":ti,ab,kw,de OR "TBS":ti,ab,kw,de OR "Synchronised transcranial magnetic stimulation":ti,ab,kw,de OR "sTMS":ti,ab,kw,de |
| #15 | "transcranial direct current stimulation"/exp |
| #16 | "Transcranial Direct Current Stimulation":ti,ab,kw,de OR "tDCS":ti,ab,kw,de OR "Cathodal Stimulation Transcranial Direct Current Stimulation":ti,ab,kw,de OR "Cathodal Stimulation tDCS":ti,ab,kw,de OR "Cathodal Stimulation tDCSs":ti,ab,kw,de OR "Stimulation tDCS, Cathodal":ti,ab,kw,de OR "Stimulation tDCSs, Cathodal":ti,ab,kw,de OR "tDCS, Cathodal Stimulation":ti,ab,kw,de OR "tDCSs, Cathodal Stimulation":ti,ab,kw,de OR "Transcranial Random Noise Stimulation":ti,ab,kw,de OR "Transcranial Alternating Current Stimulation":ti,ab,kw,de OR "Transcranial Electrical Stimulation":ti,ab,kw,de OR "Electrical Stimulation, Transcranial":ti,ab,kw,de OR "Electrical Stimulations, Transcranial":ti,ab,kw,de OR "Stimulation, Transcranial Electrical":ti,ab,kw,de OR "Stimulations, Transcranial Electrical":ti,ab,kw,de OR "Transcranial Electrical Stimulations":ti,ab,kw,de OR "Anodal Stimulation Transcranial Direct Current Stimulation":ti,ab,kw,de OR "Anodal Stimulation tDCS":ti,ab,kw,de OR "Anodal Stimulation tDCSs":ti,ab,kw,de OR "Stimulation tDCS, Anodal":ti,ab,kw,de OR "Stimulation tDCSs, Anodal":ti,ab,kw,de OR "tDCS, Anodal Stimulation":ti,ab,kw,de OR "tDCSs, Anodal Stimulation":ti,ab,kw,de OR "Repetitive Transcranial Electrical Stimulation":ti,ab,kw,de OR "tACS":ti,ab,kw,de |
| #17  Combine | "non-invasive brain stimulation":ti,ab,kw,de OR "NIBS":ti,ab,kw,de OR "Non-invasive neurostimulation":ti,ab,kw,de OR "Neuro stimulation":ti,ab,kw,de OR "Cortical stimulation":ti,ab,kw,de OR "brain depth stimulation"/exp OR "deep brain stimulator"/exp OR "Deep Brain Stimulation":ti,ab,kw,de OR "Brain Stimulations, Deep":ti,ab,kw,de OR "Deep Brain Stimulations":ti,ab,kw,de OR "Stimulation, Deep Brain":ti,ab,kw,de OR "Stimulations, Deep Brain":ti,ab,kw,de OR "Brain Stimulation, Deep":ti,ab,kw,de OR "Electrical Stimulation of the Brain":ti,ab,kw,de OR "Electrical brain stimulation":ti,ab,kw,de OR "EBS":ti,ab,kw,de OR "Brain stimulation":ti,ab,kw,de OR "brain excitation":ti,ab,kw,de OR "brain stimulation":ti,ab,kw,de OR "brain stimulus":ti,ab,kw,de OR "deep brain stimulation":ti,ab,kw,de OR "excitation, brain":ti,ab,kw,de OR "deep brain electrical stimulation system":ti,ab,kw,de OR "deep brain stimulation device":ti,ab,kw,de OR "deep brain stimulation system":ti,ab,kw,de OR "transcranial magnetic stimulation"/exp OR "Transcranial Magnetic Stimulation":ti,ab,kw,de OR "Magnetic Stimulation, Transcranial":ti,ab,kw,de OR "Magnetic Stimulations, Transcranial":ti,ab,kw,de OR "Stimulation, Transcranial Magnetic":ti,ab,kw,de OR "Stimulations, Transcranial Magnetic":ti,ab,kw,de OR "Transcranial Magnetic Stimulations":ti,ab,kw,de OR "Transcranial Magnetic Stimulation, Single Pulse":ti,ab,kw,de OR "Transcranial Magnetic Stimulation, Paired Pulse":ti,ab,kw,de OR "Transcranial Magnetic Stimulation, RepetitiveTMS":ti,ab,kw,de OR "repetitive transcranial magnetic stimulation":ti,ab,kw,de OR "rTMS":ti,ab,kw,de OR "TMS":ti,ab,kw,de OR "repetitive transcranial magnetic stimulation":ti,ab,kw,de OR "rTMS":ti,ab,kw,de OR "Accelerated transcranial magnetic stimulation":ti,ab,kw,de OR "aTMS":ti,ab,kw,de OR "Priming transcranial magnetic stimulation":ti,ab,kw,de OR "pTMS":ti,ab,kw,de OR "Deep transcranial magnetic stimulation":ti,ab,kw,de OR "dTMS":ti,ab,kw,de OR "Theta burst stimulation":ti,ab,kw,de OR "TBS":ti,ab,kw,de OR "Synchronised transcranial magnetic stimulation":ti,ab,kw,de OR "sTMS":ti,ab,kw,de OR "transcranial direct current stimulation"/exp OR "Transcranial Direct Current Stimulation":ti,ab,kw,de OR "tDCS":ti,ab,kw,de OR "Cathodal Stimulation Transcranial Direct Current Stimulation":ti,ab,kw,de OR "Cathodal Stimulation tDCS":ti,ab,kw,de OR "Cathodal Stimulation tDCSs":ti,ab,kw,de OR "Stimulation tDCS, Cathodal":ti,ab,kw,de OR "Stimulation tDCSs, Cathodal":ti,ab,kw,de OR "tDCS, Cathodal Stimulation":ti,ab,kw,de OR "tDCSs, Cathodal Stimulation":ti,ab,kw,de OR "Transcranial Random Noise Stimulation":ti,ab,kw,de OR "Transcranial Alternating Current Stimulation":ti,ab,kw,de OR "Transcranial Electrical Stimulation":ti,ab,kw,de OR "Electrical Stimulation, Transcranial":ti,ab,kw,de OR "Electrical Stimulations, Transcranial":ti,ab,kw,de OR "Stimulation, Transcranial Electrical":ti,ab,kw,de OR "Stimulations, Transcranial Electrical":ti,ab,kw,de OR "Transcranial Electrical Stimulations":ti,ab,kw,de OR "Anodal Stimulation Transcranial Direct Current Stimulation":ti,ab,kw,de OR "Anodal Stimulation tDCS":ti,ab,kw,de OR "Anodal Stimulation tDCSs":ti,ab,kw,de OR "Stimulation tDCS, Anodal":ti,ab,kw,de OR "Stimulation tDCSs, Anodal":ti,ab,kw,de OR "tDCS, Anodal Stimulation":ti,ab,kw,de OR "tDCSs, Anodal Stimulation":ti,ab,kw,de OR "Repetitive Transcranial Electrical Stimulation":ti,ab,kw,de OR "tACS":ti,ab,kw,de |
| #18  Combine | #7 AND #17 |

Appendix Table 4. Cochrane Library database search strategy

| **Search** | **Search terms** |
| --- | --- |
| #1 | [mh "Alcoholism"] |
| #2 | "Alcoholism":ti,ab,kw OR "Alcohol Dependence":ti,ab,kw OR "Dependence, Alcohol":ti,ab,kw OR "Alcohol Addiction":ti,ab,kw OR "Addiction, Alcohol":ti,ab,kw OR "Alcoholic Intoxication, Chronic":ti,ab,kw OR "Chronic Alcoholic Intoxication":ti,ab,kw OR "Intoxication, Chronic Alcoholic":ti,ab,kw OR "Alcohol Use Disorder":ti,ab,kw OR "Alcohol Use Disorders":ti,ab,kw OR "Use Disorder, Alcohol":ti,ab,kw OR "Use Disorders, Alcohol":ti,ab,kw OR "Alcohol Abuse":ti,ab,kw OR "Abuse, Alcohol":ti,ab,kw |
| #3 | [mh "Substance-Related Disorders"] |
| #4 | "Substance-Related Disorders":ti,ab,kw OR "Drug Abuse":ti,ab,kw OR "Abuse, Drug":ti,ab,kw OR "Drug Dependence":ti,ab,kw OR "Dependence, Drug":ti,ab,kw OR "Drug Addiction":ti,ab,kw OR "Addiction, Drug":ti,ab,kw OR "Substance Use Disorders":ti,ab,kw OR "Disorder, Substance Use":ti,ab,kw OR "Substance Use Disorder":ti,ab,kw OR "Drug Use Disorders":ti,ab,kw OR "Disorder, Drug Use":ti,ab,kw OR "Drug Use Disorder":ti,ab,kw OR "Organic Mental Disorders, Substance-Induced":ti,ab,kw OR "Organic Mental Disorders, Substance Induced":ti,ab,kw OR "Substance Abuse":ti,ab,kw OR "Abuse, Substance":ti,ab,kw OR "Abuses, Substance":ti,ab,kw OR "Substance Abuses":ti,ab,kw OR "Substance Dependence":ti,ab,kw OR "Dependence, Substance":ti,ab,kw OR "Substance Addiction":ti,ab,kw OR "Addiction, Substance":ti,ab,kw OR "Prescription Drug Abuse":ti,ab,kw OR "Abuse, Prescription Drug":ti,ab,kw OR "Drug Abuse, Prescription":ti,ab,kw OR "Drug Habituation":ti,ab,kw OR "Habituation, Drug":ti,ab,kw |
| #5 | [mh "Alcoholics"] |
| #6 | "Alcoholics":ti,ab,kw OR "Alcoholic":ti,ab,kw OR "Skid Row Alcoholics":ti,ab,kw OR "Alcoholic, Skid Row":ti,ab,kw OR "Alcoholics, Skid Row":ti,ab,kw OR "Skid Row Alcoholic":ti,ab,kw |
| #7 | [mh "Alcohol-Related Disorders"] |
| #8 | "Alcohol-Related Disorders":ti,ab,kw OR "Alcohol Related Disorders":ti,ab,kw OR "Alcohol-Related Disorder":ti,ab,kw OR "Disorder, Alcohol-Related":ti,ab,kw OR "Disorders, Alcohol-Rel":ti,ab,kw |
| #9  Combine | {OR #1-#8} |
| #10 | "non-invasive brain stimulation":ti,ab,kw OR "NIBS":ti,ab,kw OR "Non-invasive neurostimulation":ti,ab,kw OR "Neuro stimulation":ti,ab,kw OR "Cortical stimulation":ti,ab,kw |
| #11 | [mh "Deep Brain Stimulation"] |
| #12 | "Deep Brain Stimulation":ti,ab,kw OR "Brain Stimulations, Deep":ti,ab,kw OR "Deep Brain Stimulations":ti,ab,kw OR "Stimulation, Deep Brain":ti,ab,kw OR "Stimulations, Deep Brain":ti,ab,kw OR "Brain Stimulation, Deep":ti,ab,kw OR "Electrical Stimulation of the Brain":ti,ab,kw OR "Electrical brain stimulation":ti,ab,kw OR "EBS":ti,ab,kw OR "Brain stimulation":ti,ab,kw |
| #13 | [mh "Transcranial Magnetic Stimulation"] |
| #14 | "Transcranial Magnetic Stimulation":ti,ab,kw OR "Magnetic Stimulation, Transcranial":ti,ab,kw OR "Magnetic Stimulations, Transcranial":ti,ab,kw OR "Stimulation, Transcranial Magnetic":ti,ab,kw OR "Stimulations, Transcranial Magnetic":ti,ab,kw OR "Transcranial Magnetic Stimulations":ti,ab,kw OR "Transcranial Magnetic Stimulation, Single Pulse":ti,ab,kw OR "Transcranial Magnetic Stimulation, Paired Pulse":ti,ab,kw OR "Transcranial Magnetic Stimulation, RepetitiveTMS":ti,ab,kw OR "repetitive transcranial magnetic stimulation":ti,ab,kw OR "rTMS":ti,ab,kw OR "TMS":ti,ab,kw OR "repetitive transcranial magnetic stimulation":ti,ab,kw OR "rTMS":ti,ab,kw |
| #15 | "Accelerated transcranial magnetic stimulation":ti,ab,kw OR "aTMS":ti,ab,kw OR "Priming transcranial magnetic stimulation":ti,ab,kw OR "pTMS":ti,ab,kw OR "Deep transcranial magnetic stimulation":ti,ab,kw OR "dTMS":ti,ab,kw OR "Theta burst stimulation":ti,ab,kw OR "TBS":ti,ab,kw OR "Synchronised transcranial magnetic stimulation":ti,ab,kw OR "sTMS":ti,ab,kw |
| #16 | [mh "Transcranial Direct Current Stimulation"] |
| #17 | "Transcranial Direct Current Stimulation":ti,ab,kw OR "tDCS":ti,ab,kw OR "Cathodal Stimulation Transcranial Direct Current Stimulation":ti,ab,kw OR "Cathodal Stimulation tDCS":ti,ab,kw OR "Cathodal Stimulation tDCSs":ti,ab,kw OR "Stimulation tDCS, Cathodal":ti,ab,kw OR "Stimulation tDCSs, Cathodal":ti,ab,kw OR "tDCS, Cathodal Stimulation":ti,ab,kw OR "tDCSs, Cathodal Stimulation":ti,ab,kw OR "Transcranial Random Noise Stimulation":ti,ab,kw OR "Transcranial Alternating Current Stimulation":ti,ab,kw OR "Transcranial Electrical Stimulation":ti,ab,kw OR "Electrical Stimulation, Transcranial":ti,ab,kw OR "Electrical Stimulations, Transcranial":ti,ab,kw OR "Stimulation, Transcranial Electrical":ti,ab,kw OR "Stimulations, Transcranial Electrical":ti,ab,kw OR "Transcranial Electrical Stimulations":ti,ab,kw OR "Anodal Stimulation Transcranial Direct Current Stimulation":ti,ab,kw OR "Anodal Stimulation tDCS":ti,ab,kw OR "Anodal Stimulation tDCSs":ti,ab,kw OR "Stimulation tDCS, Anodal":ti,ab,kw OR "Stimulation tDCSs, Anodal":ti,ab,kw OR "tDCS, Anodal Stimulation":ti,ab,kw OR "tDCSs, Anodal Stimulation":ti,ab,kw OR "Repetitive Transcranial Electrical Stimulation":ti,ab,kw OR "tACS":ti,ab,kw |
| #18  Combine | {OR #10-#17} |
| #19  Combine | #9 AND #18 |

Appendix Table 5. PsycINFO database search strategy

| **Search** | **Search terms** |
| --- | --- |
| #1 | "Alcoholism" OR "Alcohol Dependence" OR "Dependence, Alcohol" OR "Alcohol Addiction" OR "Addiction, Alcohol" OR "Alcoholic Intoxication, Chronic" OR "Chronic Alcoholic Intoxication" OR "Intoxication, Chronic Alcoholic" OR "Alcohol Use Disorder" OR "Alcohol Use Disorders" OR "Use Disorder, Alcohol" OR "Use Disorders, Alcohol" OR "Alcohol Abuse" OR "Abuse, Alcohol" OR ”Substance-Related Disorders" OR "Drug Abuse" OR "Abuse, Drug" OR "Drug Dependence" OR "Dependence, Drug" OR "Drug Addiction" OR "Addiction, Drug" OR "Substance Use Disorders" OR "Disorder, Substance Use" OR "Substance Use Disorder" OR "Drug Use Disorders" OR "Disorder, Drug Use" OR "Drug Use Disorder" OR "Organic Mental Disorders, Substance-Induced" OR "Substance Abuse" OR "Abuse, Substance" OR "Abuses, Substance" OR "Substance Abuses" OR "Substance Dependence" OR "Dependence, Substance" OR "Substance Addiction" OR "Addiction, Substance" OR "Prescription Drug Abuse" OR "Abuse, Prescription Drug" OR "Drug Abuse, Prescription" OR "Drug Habituation" OR "Habituation, Drug" OR "Alcoholics" OR "Alcoholics" OR "Alcoholic" OR "Skid Row Alcoholics" OR "Alcoholic, Skid Row" OR "Alcoholics, Skid Row" OR "Skid Row Alcoholic" OR "Alcohol Related Disorders" OR "Alcohol-Related Disorder" OR "Disorder, Alcohol-Related" OR "Disorders, Alcohol-Rel" |
| #2 | "non-invasive brain stimulation" OR "NIBS" OR "Non-invasive neurostimulation" OR "Neuro stimulation" OR "Cortical stimulation" OR "Deep Brain Stimulation" OR "Brain Stimulations, Deep" OR "Deep Brain Stimulations" OR "Stimulation, Deep Brain" OR "Stimulations, Deep Brain" OR "Brain Stimulation, Deep" OR "Electrical Stimulation of the Brain" OR "Electrical brain stimulation" OR "EBS" OR "Brain stimulation" OR "Transcranial Magnetic Stimulation" OR "Magnetic Stimulation, Transcranial" OR "Magnetic Stimulations, Transcranial" OR "Stimulation, Transcranial Magnetic" OR "Stimulations, Transcranial Magnetic" OR "Transcranial Magnetic Stimulations" OR "Transcranial Magnetic Stimulation, Single Pulse" OR "Transcranial Magnetic Stimulation, Paired Pulse" OR "Transcranial Magnetic Stimulation, Repetitive TMS" OR “repetitive transcranial magnetic stimulation" OR "rTMS" OR "TMS" OR "repetitive transcranial magnetic stimulation" OR "Accelerated transcranial magnetic stimulation" OR "aTMS" OR "Priming transcranial magnetic stimulation" OR "pTMS" OR "Deep transcranial magnetic stimulation" OR "dTMS" OR "Theta burst stimulation" OR "TBS" OR "Synchronised transcranial magnetic stimulation" OR "sTMS" OR "Transcranial Direct Current Stimulation" OR "tDCS" OR "Cathodal Stimulation Transcranial Direct Current Stimulation" OR "Cathodal Stimulation tDCS" OR "Cathodal Stimulation tDCSs" OR "Stimulation tDCS, Cathodal" OR "Stimulation tDCSs, Cathodal" OR "tDCS, Cathodal Stimulation" OR "tDCSs, Cathodal Stimulation" OR "Transcranial Random Noise Stimulation" OR "Transcranial Alternating Current Stimulation" OR "Transcranial Electrical Stimulation" OR "Electrical Stimulation, Transcranial" OR "Electrical Stimulations, Transcranial" OR "Stimulation, Transcranial Electrical" OR "Stimulations, Transcranial Electrical" OR "Transcranial Electrical Stimulations" OR "Anodal Stimulation Transcranial Direct Current Stimulation" OR "Anodal Stimulation tDCS" OR "Anodal Stimulation tDCSs" OR "Stimulation tDCS, Anodal" |
| #3  Combine | #1 AND #2 |

Appendix Table 6. Risk of bias assessment criteria

| Domain | Risk classification | Criteria |
| --- | --- | --- |
| Sequence generation | Low risk | Randomization described, such as flipping a coin, using computer software, etc. |
|  | Unclear risk | Randomization method is not described, or it is unclear if the method is appropriate |
|  | High risk | Incorrect randomization method applied |
| Allocation concealment | Low risk | Describe the method of concealment, such as using envelopes, computer databases, etc. |
|  | Unclear risk | Concealment method not described |
|  | High risk | Allocation was not applied |
| Blinding of performance and personnel | Low risk | The blinding must be described in detail. Integrity of subject blinding was successful. It is described that the operator did not communicate with staff (or was blinded) |
|  | Unclear risk | Blinding was not properly described. The integrity of the blinding was not tested. Communication between practitioner and staff (or blinding) is not described |
|  | High risk | Blinding was not applied. Communication between operator and staff was present |
| Blinding of outcome assessment | Low risk | Describes that the rater was blinded |
|  | Unclear risk | Does not describe whether the rater was blinded or not |
|  | High risk | Rater was not blinded to the assigned intervention |
| Incomplete outcome data | Low risk | Attrition or dropouts was adequately reported with valid reasons for missing data |
|  | Unclear risk | Attrition was not adequately described |
|  | High risk | Attrition rates were not balanced between groups. Statistical Analyses was only performed on patients who complied to the protocol. |
| Selective outcome reporting | Low risk | All outcomes was reported |
|  | Unclear risk | Not all outcomes were reported, but primary outcomes were reported |
|  | High risk | Results of at least one primary outcome was not reported |
| Other bias | Low risk | No other bias detected |
|  | Unclear risk | There may be a risk of bias, but there is either insufficient information to assess whether an important risk of bias exists or insufficient rationale or evidence that an identified problem will introduce bias |
|  | High risk | Bias due to problems not covered elsewhere in the risk of bias assessment table |
| Overall risk of bias | Low risk | low risk in all domains or 1 domain unclear |
|  | Unclear risk | At least two domains with unclear risk of bias |
|  | High risk | At least one domain with high risk of bias |

Appendix Table 7. Types of NIBS

| Characteristic | N.units | N.patients |
| --- | --- | --- |
| a-F3+c-F4 tDCS | 1 | 13 |
| a-F3-tDCS | 3 | 83 |
| a-F4+c-F3 tDCS | 7 | 161 |
| a-right IFG-tDCS | 1 | 15 |
| DLPFC-dTMS | 1 | 5 |
| FP1-cTBS | 1 | 24 |
| F3-iTBS | 1 | 8 |
| F4-rTMS | 4 | 87 |
| IC-rTMS | 1 | 23 |
| mPFC-dTMS | 2 | 32 |
| sham | 22 | 409 |

Appendix Table 8. Assessment of the risk of bias for studies

| First Author  (Year) | D1 | D2 | D3 | D4 | D5 | D6 | D7 | Overall |
| --- | --- | --- | --- | --- | --- | --- | --- | --- |
| Boggio et al.  (2008) | Low | Unclear | Unclear | Low | Low | Low | Low | Unclear |
| Mishra et al.  (2010) | High | Unclear | Unclear | High | Low | Low | Low | High |
| Herremans et al.  (2012) | Low | Unclear | Unclear | High | Low | Low | Low | High |
| Nakamura-Palacios et al. (2012) | Unclear | Unclear | Unclear | High | Unclear | Low | Low | High |
| Herremans et al.  (2013) | Low | Low | Unclear | High | Low | Low | Low | High |
| Klauss et al.  (2014) | Low | Low | Low | Low | Low | Low | Low | Low |
| Ceccanti et al.  (2015) | Low | Low | Low | Low | Low | Low | Low | Low |
| den-Uyl et al.  (2015) | Unclear | Unclear | Unclear | Low | Low | Low | Low | Unclear |
| Herremans et al.  (2015) | Low | Unclear | Low | High | Low | Low | Low | High |
| Wietschorke et al.  (2016) | Unclear | Unclear | Unclear | Low | Low | Low | Low | Unclear |
| Addolorato et al.  (2017) | Low | Unclear | Unclear | Low | Low | Low | Low | Unclear |
| den-Uyl et al.  (2018) | Low | Unclear | Low | Unclear | Low | Low | Low | Unclear |
| Hanlon et al.  (2018) | Unclear | Unclear | Low | High | Low | Low | Low | High |
| Klauss et al.  (2018) | Low | Low | Low | Low | Low | Low | Low | Low |
| Holla et al.  (2020) | Low | Low | Low | Low | Low | Low | Low | Low |
| Perini et al.  (2020) | Low | Low | Low | Low | Low | Low | Low | Low |
| Vanderhasselt et al.  (2020) | Unclear | Low | Low | Low | Low | Low | Low | Low |
| Harel et al.  (2022) | Low | Unclear | Low | Low | Unclear | Low | Low | Unclear |
| Padula et al.  (2023) | Low | Low | Unclear | Unclear | Low | Low | Low | Unclear |
| Patil et al.  (2024) | Low | Unclear | Unclear | High | Low | Low | Low | High |

Domains : D1, Sequence generation; D2, Allocation concealment; D3, Blinding of performance and personnel; D4, Blinding of outcome assessment; D5, Incomplete outcome data; D6, Selective outcome reporting; D7, Other bias

Appendix Table 9. Assessment of quality of evidence

| **Certainty assessment** | | | | | | | **№ of patients** | | **Effect** | | **Certainty** | **Importance** |
| --- | --- | --- | --- | --- | --- | --- | --- | --- | --- | --- | --- | --- |
| **№ of studies** | **Study design** | **Risk of bias** | **Inconsistency** | **Indirectness** | **Imprecision** | **Other considerations** | **[intervention] NIBS** | **[comparison] Sham** | **Relative (95% CI)** | **Absolute (95% CI)** |  |  |
| **Change in alcohol craving severity** | | | | | | | | | | | | |
| 22 | randomised trials | serious | not serious | not serious | not serious | none | 451 | 409 | - | SMD **0.21 SD lower** (0.37 lower to 0.04 lower) | ⨁⨁⨁◯ Moderate | IMPORTANT |
| **Rate of adverse events** | | | | | | | | | | | | |
| 9 | randomised trials | serious | not serious | not serious | serious | none | 54/205 (26.3%) | 34/175 (19.4%) | **OR 1.49** (0.83 to 2.67) | **70 more per 1,000** (from 28 fewer to 197 more) | ⨁⨁◯◯ Low | IMPORTANT |

**CI:** confidence interval; **OR:** odds ratio; **SMD:** standardised mean difference
